# The Effects of Electrical and Optical Stimulation of Midbrain Dopaminergic Neurons on Rat 50-kHz Ultrasonic Vocalizations

**DOI:** 10.3389/fnbeh.2015.00331

**Published:** 2015-12-08

**Authors:** Tina Scardochio, Ivan Trujillo-Pisanty, Kent Conover, Peter Shizgal, Paul B. S. Clarke

**Affiliations:** ^1^Department of Pharmacology and Therapeutics, Neuropsychopharmacology, McGill UniversityMontreal, QC, Canada; ^2^Department of Psychology, Center for Studies in Behavioral Neurobiology, Concordia UniversityMontreal, QC, Canada

**Keywords:** phasic dopamine, ultrasonic vocalizations, nucleus accumbens, midbrain dopaminergic neurons, optogenetics, fast-scan cyclic voltammetry

## Abstract

**Rationale:** Adult rats emit ultrasonic vocalizations (USVs) at around 50-kHz; these commonly occur in contexts that putatively engender positive affect. While several reports indicate that dopaminergic (DAergic) transmission plays a role in the emission of 50-kHz calls, the pharmacological evidence is mixed. Different modes of dopamine (DA) release (i.e., tonic and phasic) could potentially explain this discrepancy.

**Objective:** To investigate the potential role of *phasic* DA release in 50-kHz call emission.

**Methods:** In Experiment 1, USVs were recorded in adult male rats following unexpected electrical stimulation of the medial forebrain bundle (MFB). In parallel, phasic DA release in the nucleus accumbens (NAcc) was recorded using fast-scan cyclic voltammetry. In Experiment 2, USVs were recorded following response-contingent or non-contingent optogenetic stimulation of midbrain DAergic neurons. Four 20-s schedules of optogenetic stimulation were used: fixed-interval, fixed-time, variable-interval, and variable-time.

**Results:** Brief electrical stimulation of the MFB increased both 50-kHz call rate and phasic DA release in the NAcc. During optogenetic stimulation sessions, rats initially called at a high rate comparable to that observed following reinforcers such as psychostimulants. Although optogenetic stimulation maintained reinforced responding throughout the 2-h session, the call rate declined to near zero within the first 30 min. The trill call subtype predominated following both electrical and optical stimulation.

**Conclusion:** The occurrence of electrically-evoked 50-kHz calls, time-locked to phasic DA (Experiment 1), provides correlational evidence supporting a role for phasic DA in USV production. However, in Experiment 2, the temporal dissociation between calling and optogenetic stimulation of midbrain DAergic neurons suggests that phasic mesolimbic DA release is not sufficient to produce 50-kHz calls. The emission of the trill subtype of 50-kHz calls potentially provides a marker distinguishing positive affect from positive reinforcement.

## Introduction

Adult rats produce two categories of ultrasonic vocalizations (USVs; Portfors, 2007; Clarke and Wright, 2015): 22-kHz (range: 20–30 kHz) and 50-kHz (30–90 kHz). Calls in both categories are thought to play a role in communication (for review see: Seffer et al., 2014). In addition to their proposed communicative role, the 22- and 50-kHz call categories appear to reflect negative and positive affective states, respectively (Knutson et al., 2002). Thus, while 22-kHz calls are commonly associated with aversive situations (Litvin et al., 2007; Mahler et al., 2013), 50-kHz calls have been detected during a variety of rewarding events, such as rough-and-tumble play and administration of psychostimulant drugs (Burgdorf et al., 2001; Panksepp and Burgdorf, 2003; Wright et al., 2010).

Several neurotransmitters appear to play a role in the emission of 50-kHz USVs in adult rats (Fu and Brudzynski, 1994; Panksepp and Burgdorf, 2000; Wintink and Brudzynski, 2001; Fendt et al., 2006; Burgdorf et al., 2007; Arnold et al., 2010; Sadananda et al., 2012; Wright et al., 2012, 2013; Manduca et al., 2014; Wöhr et al., 2015). Here, dopamine (DA) has received particular attention, given its well-established role in motivation and reward (Bromberg-Martin et al., 2010; Ikemoto, 2010; Covey et al., 2014; Ranaldi, 2014). A variety of dopaminergic (DAergic) manipulations alter the rate at which adult rats emit 50-kHz calls (Burgdorf et al., 2000, 2001, 2007; Williams and Undieh, 2010; Brudzynski et al., 2012; Simola et al., 2014); in particular, DAergic transmission in the nucleus accumbens (NAcc) appears both necessary and sufficient for call emission, as evidenced by studies using DA-targeted lesions and intracerebral microinjection of DAergic drugs (Burgdorf et al., 2001, 2007). Accordingly, we and others have found that both amphetamine (AMPH)-induced and spontaneous 50-kHz vocalizations are profoundly inhibited by systemically-administered D1 and D2 antagonists (Scardochio and Clarke, 2013; Wright et al., 2013; Wöhr et al., 2015). However, some DAergic drugs have produced unexpected effects after systemic administration: (1) direct DAergic agonists *inhibited* 50-kHz calling across a wide dose range (Scardochio and Clarke, 2013), and (2) the DA transporter blocker GBR 12909 failed to mimic AMPH's stimulatory effect on 50-kHz call emission, even when combined with a noradrenaline transporter blocker (Wright et al., 2013).

These apparently conflicting findings might reflect differential drug effects on two distinct modes of DA transmission: tonic and phasic. In the absence of salient stimuli, midbrain DAergic neurons display a tonic pacemaker-like activity, maintaining a stable and low DA extracellular concentration (“tone”) in terminal structures such as the NAcc (Grace and Bunney, 1984). Salient stimuli such as unexpected rewards induce neuronal burst firing, resulting in phasic DA release associated with a rapid and transient increase in extracellular DA concentrations (Schultz et al., 1997; Wightman and Robinson, 2002; Redgrave et al., 2008). Several observations suggest that 50-kHz call emission may be associated with *phasic* DA release. Notably, 50-kHz vocalizations have been evoked by several manipulations that have been shown to increase phasic DA release: experimenter-delivered “tickling” (Hori et al., 2013), playback of 50-kHz vocalizations (Willuhn et al., 2014) and the presence of a conspecific receiving reward (Kashtelyan et al., 2014). Rats will also emit 50-kHz calls in anticipation of electrical stimulation of the medial forebrain bundle (MFB) that would be expected to increase phasic DA release in terminal areas such as the NAcc (Burgdorf et al., 2000). If phasic DA transmission promotes USV emission, this could also reconcile several pharmacological findings: (1) AMPH and cocaine reliably induced 50-kHz calling and increased phasic DA release (Cheer et al., 2007; Wright et al., 2010, 2013; Willuhn et al., 2012; Daberkow et al., 2013; Covey et al., 2014), (2) tonic activation of postsynaptic receptors by selective DA receptor agonists inhibited spontaneous calling (Scardochio and Clarke, 2013), and (3) the DAT blocker GBR 12909, which did not increase USV emission (Wright et al., 2013), is expected to increase both tonic and phasic DA signaling (Reith et al., 1997; Budygin et al., 2000; Owesson-White et al., 2012), the former effect potentially masking the latter.

The aim of the present study was therefore to test whether phasic DA release events drive 50-kHz calls in adult rats. Previously, Burgdorf et al. (2007) showed that electrical stimulation of the MFB evoked 50-kHz call emission and that these calls were decreased by the DA antagonist flupenthixol. However, flupenthixol has non-DAergic effects, notably on 5-HT receptors (Kühn et al., 2000), and in addition phasic DA release was inferred rather than measured. Therefore, to directly assess the involvement of phasic DA release, our first experiment asked whether electrical stimulation of the MFB would elicit USVs using parameters that evoked phasic DA release events detected using fast-scan cyclic voltammetry. The second experiment investigated whether phasic DA activity was *sufficient* to induce 50-kHz calls. Here, we recorded USVs during optogenetic stimulation that was designed to selectively activate midbrain DAergic neurons. Two parameters of reinforcement were investigated: (1) expected vs. unexpected stimulation to test for anticipatory calling and (2) contingent vs. non-contingent stimulation to allow for comparison with previous studies using electrical stimulation.

## Methods

### Acquisition and identification of ultrasonic vocalizations

The testing procedure and acoustic analysis were in part, as previously described (Wright et al., 2010). For the initial amphetamine screen, clear Plexiglas™ experimental boxes (ENV-007CT, Med Associates, St. Albans, VT) were used for testing and each was enclosed in a separate melamine compartment lined with sound attenuating acoustic foam (Primacoustic, Port Coquitlam, BC). For the optogenetic and fast-scan cyclic voltammetry (FSCV) experiments, Clear Plexiglas™ experimental boxes (optogenetic: ENV-007CT, Med Associates, St. Albans, VT, FSCV: made in-house) were also used but sound attenuating acoustic foam was not utilized. Condenser ultrasound microphones (CM16/CMPA, Avisoft Bioacoustics, Berlin, Germany) were located at the top center of each test box, 30–60 cm from the rat. Microphone signals were delivered to an UltraSoundGate 416H data acquisition device (Avisoft Bioacoustics, Glienicke, Germany) with a sampling rate of 250 kHz and 16-bit resolution. Avisoft SASLab Pro software (version 5.1.14, Avisoft Bioacoustics, Berlin, Germany) was used for acoustical analysis. Spectrograms were created with a fast Fourier transform (length 512 points, overlap 75%, FlatTop window, 100% frame size) yielding a frequency resolution of 490 Hz and a time resolution of 0.5 ms. Calls were selected manually from spectrograms by an individual masked to treatment conditions. Call rate was defined as the total number of 50-kHz calls per 5 min, unless otherwise noted. All calls were categorized into one of 14 subtypes (see examples in Supplementary Figure 1), as defined by Wright et al. (2010), plus two additional categories that are rarely observed (~1% of calls): “unclassifiable” (call was not loud enough, or noise was present, which prevented accurate subtyping) and “miscellaneous” (call was visible but did not clearly fit one of the 14 subtypes).

### Statistics

Data were analyzed using commercial software (Systat v11, SPSS, Chicago, IL; GraphPad Software, La Jolla, CA). Except during the MFB stimulation experiment (see Section Results below), USVs between 20- and 30-kHz were rarely observed and were not analyzed statistically; otherwise, all USVs refer to the 50-kHz subtype. Nonparametric tests were used when the data suggested that parametric test assumptions (e.g., variance homogeneity) were violated. Multiple vehicle conditions were compared by Friedman's nonparametric analysis of variance. Specific comparisons were performed using Wilcoxon signed-rank tests or Sign tests. For all tests, a two-tailed *p*-value < 5% was considered significant. For USV data, *n* = number of rats and for the electrochemical data, *n* = number of electrodes (see Section Voltammetric Data Analysis below).

## Experiment 1 electrical stimulation of the medial forebrain bundle and FSCV recordings

### Subjects

Eight experimentally naïve male Long-Evans rats (Charles River Laboratories, St. Constant, Quebec, Canada) were used, weighing 359–420 g at surgery. Subjects were housed two per cage before surgery and singly-housed after surgery. Home cages were kept in a temperature- and humidity-controlled colony room (20–22°C, 50–60%) with laboratory grade Sani-Chips bedding (Harlan Laboratories, Indianapolis, IN). Rats were kept on a reverse 12:12 h light/dark cycle, with lights off at 0730 h. Behavioral testing took place between 0800 and 1300 h. Food and water were available *ad libitum*, except during testing. Subjects were handled once daily for 5 min, for 4 days prior to the first experimental day. All procedures were approved by the McGill Animal Care Committee in accordance with the guidelines of the Canadian Council on Animal Care.

### Electrochemical microsensor and reference electrode fabrication

Dopamine microsensors (i.e., working electrodes) and reference electrodes for chronic implantation were fabricated as previously described (Clark et al., 2010). Briefly, a single carbon fiber (grade 34–700; Goodfellow Corporation, Caraopolis, PA, USA) was threaded through a fused silica shaft (Polymicro Technologies, Phoenix, AZ, USA) while submerged in 2-propanol. With a short length (~17 mm) of carbon fiber protruding, two-component epoxy (Lepage Speed Set Epoxy™) was applied to one end and allowed to dry for 3–5 h. Next, silver epoxy (MG Chemicals, Allied Electronics, Fort Worth, TX, USA) was applied to the other end of the silica shaft in order to secure electrical contact between the carbon fiber and a gold-plated PCB socket connector (Newark Element 14 #23K7802, Chicago, IL, USA). The silver epoxy was left to dry overnight, then coated with the two-component epoxy. The protruding length of carbon fiber was trimmed to 150–200 μm.

Reference electrodes were made using silver wire (A-M systems, bare: 0.010″, coated: 0.013″) cut into lengths of ~1 cm. A small drop of silver epoxy was applied to the open end of a nickel-plated brass pin (Newark Element 14 #82K7794, Chicago, IL, USA) and a single piece of uncoated silver wire was inserted. Once dry, the silver epoxy was covered with two-component epoxy and the protruding silver wire was trimmed to ~3 mm. The day before surgery, this wire was soaked in 10.2% sodium hypochlorite overnight, creating a silver/silver chloride (Ag/AgCl) surface interface.

### Voltammetry surgery

All surgical procedures followed aseptic technique. Rats were anesthetized with isoflurane (5% induction, 2–2.5% maintenance, AErrane, Baxter). The scalp was shaved before the rat was placed in a stereotaxic frame. Polyvinyl alcohol (1% w/v, HypoTears, Novartis) was applied to the eyes, and a non-steroidal anti-inflammatory analgesic (carprofen, 5 mg/kg) and 0.9% sterile saline (2 mL) were administered subcutaneously (SC). The scalp was incised along the midline after topical application of Baxedin™antiseptic (0.05% w/v chlorhexidine gluconate + 4% v/v isopropyl alcohol) and local anesthetic (50:50 v/v mixture of 2% lidocaine and 0.5% bupivicaine). Skull holes were drilled and cleared of dura mater above the NAcc core (2.0 mm lateral and 1.7 mm rostral to bregma) and shell (0.9 mm lateral and 1.5 mm rostral to bregma); each rat received one microsensor implanted in each hemisphere (total of two microsensors per rat). Next, a skull hole was drilled in order to position the stimulating electrode (Plastics One, MS303/2-A/SP) above the medial forebrain bundle (MFB; 1.3 mm lateral and 4.6 mm caudal to bregma). Four to five additional holes were drilled at convenient locations for a reference electrode and 3–4 anchor screws. The reference electrode was lowered 3–4 mm from the skull surface (the entire length of silver wire) and one screw was secured to the skull; both were anchored with cranioplastic cement, leaving the stimulating electrode and microsensor holes exposed. The two microsensors were then attached to the voltammetric amplifier and lowered (0.2 mm/min) into the target recording regions (7.4 mm ventral of the brain surface for core and shell of NAcc). In order to determine the ideal location for placement of the stimulating electrode, the voltammetric waveform was applied at 10 Hz and catecholamines were monitored. Next, the stimulating electrode was lowered to 7.2 mm below dura mater and electrical stimulation (24 biphasic 120 μA 60 Hz pulses, each pulse comprising a pair of 2-ms phases) was applied via an optically isolated, constant-current stimulator (A-M Systems, Sequim, Washington). If an evoked change in catecholamine concentration was not observed at the microsensor, the stimulating electrode was lowered in 0.3 mm steps until electrically-evoked catecholamine efflux was detected. The electrode was then lowered in 0.1 mm increments until catecholamine release was maximal. This usually occurred when the stimulating electrode was 8.4 or 8.7 mm ventral from the brain surface. If catecholamines were not detected at this point, the stimulating electrode was lowered to and kept at 8.7 mm. Finally, cranioplastic cement was applied to the exposed skull to secure the stimulating electrode, microsensors and the 2–3 additional screws. For post-operative pain management, subcutaneous carprofen was administered every 24 h for 4 days.

### Voltammetric data acquisition

For details of hardware and software, see (Fortin et al., 2015). Waveform generation and data acquisition were carried out by using two input/output cards (NI PCI-6052E and NI PCI-6711, National Instruments, Quebec, Canada) and software written in LabVIEW (National Instruments). Signals from chronically implanted microsensors were forwarded to the data acquisition system via a head-mounted voltammetric amplifier (current-to-voltage converter) and an electrical commutator (Crist Instrument Co Inc, MD, USA) mounted in a custom-made Faraday cage. The voltammetric amplifier comprised an operational amplifier with a feedback resistor (*R*_f_ 5 MΩ) in parallel with a 6 pF capacitor to exclude high frequencies. To filter out operational amplifier noise, additional capacitors bridged each of the power sources (+15 V, −15 V) with ground. To promote a stable background current on test days, working electrodes were cycled with the voltammetric waveform at 60 Hz for 30 min and then at 10 Hz for 15 min (Moussy and Harrison, 1994). It is not uncommon to observe a 200 mV shift in potential at reference electrodes over 2–5 days (Heien et al., 2005). This shift can be detected by the position of the Faradaic peaks within the background current. When observed, this was corrected by applying a 200 mV offset to the waveform (Heien et al., 2005). Voltammetric recordings consisted of a series of fast voltage scans, repeated at 100 ms intervals (i.e., 10 Hz). Each scan lasted 8.5 ms and comprised an ascending and descending linear (400 V/s) sweep between –0.4 and +1.3 V, applied to the microsensors in relation to the Ag/AgCl reference electrode. The potential was held at −0.4 V vs. Ag/AgCl between scans. Electrical stimulation of the MFB was used to produce phasic catecholamine release. Each stimulation train (24 biphasic pulses, each pulse comprising a pair of 2-ms phases) was spaced 5–6 min apart and the following eight stimulation parameters were used: 100 and 120 μA applied at 30 Hz, and 60, 80, 100, and 120 μA applied at 60 Hz. Voltammetric recordings were taken for 5 s before the electrical stimulation and for 10 s after. During this time, baseline USV recordings were taken for 55 s before and after each electrical stimulation.

### Voltammetric data analysis

For the chemical identification of dopamine, current during a voltammetric scan was plotted against the applied potential and the background was subtracted (LabVIEW 7.1, National Instruments), yielding a background-subtracted cyclic voltammogram (CV). Verification of the putative DA signals was performed using the “CV match” algorithm (Wightman et al., 1988; Heien et al., 2004; Clark et al., 2010) written in CV Analysis (LabView 7.1, National Instruments). Briefly, the background-subtracted CV was compared with a template voltammogram obtained from an earlier electrically-evoked (24 biphasic 120 μA 60 Hz pulses, each pulse comprising a pair of 2-ms phases) *in vivo* recording, and a correlation coefficient was obtained. The phasic event was determined to be DAergic if the correlation coefficient was ≥ 0.75 (Heien et al., 2003; Cheer et al., 2004). In addition, DA transients were required to meet this criterion for at least two consecutive scans. Peak DA currents at the oxidation potential of DA (approximately 0.65 mV) were obtained from the background-subtracted CVs. Each recording electrode (two per rat) was treated as an independent unit for the following reasons: (1) the density of DA terminals at recording sites differ (Garris et al., 1994; Peters et al., 2000), (2) DA terminals within a given brain region differ in the extent to which they are autoinhibited (Moquin and Michael, 2009), and (3) there is spatial and temporal heterogeneity of DA transmission in the ventral striatum where evoked responses are different in both amplitude and temporal profile (Wightman et al., 2007; Shu et al., 2013).

### Experimental protocol

#### Overview

Rats were tested 1 month after surgery to allow for their recovery and the stabilization of electrodes (Polikov et al., 2005; Kozai et al., 2014). Four rats survived surgery and their head caps remained intact during the subsequent month, and were thus tested further. *In vivo* testing consisted of three parts: (1) Initial amphetamine screen, (2) Test day, and (3) Characterization of the voltammetric signal.

#### Initial amphetamine screen

A significant minority of rats emit few USVs in response to various stimuli (Schwarting et al., 2007). Therefore, rats underwent an initial amphetamine screen comprising three test sessions, spaced 2 days apart (Wright et al., 2010; Scardochio and Clarke, 2013). On each day, rats (*n* = 4) were placed in test chambers for 20 min immediately after an IP injection of amphetamine (1 mg/kg, 1 mL/kg). Ultrasonic vocalizations emitted during the 12th, 14th, and 16th min of the third session were counted, and all four rats were assessed as high callers relative to previous data from our laboratory (see Section Results).

#### Test day

Testing resumed 20 days later. Rats were connected to the head-mounted voltammetric amplifier (see Data Acquisition above) and placed in the custom made test box consisting of Clear Plexiglas™ with laboratory grade Sani-Chips bedding. Half of the bedding was changed between each rat session. The electrical stimulation was not signaled by cues or demarcated by a specific point in time, and was thus unexpected.

### Characterization of *in vivo* voltammetric signals

We confirmed the dopaminergic nature of our *in vivo* voltammetry signals using three main criteria: electrochemical, pharmacological and anatomical.

#### Stimulated release

Activation of DAergic neurons by electrical stimulation of MFB can act as a positive control (Roitman et al., 2004). Here, stimulation of the MFB (24 biphasic pulses, each pulse comprising a pair of 2-ms phases, see Section Voltammetric Data Acquisition above for currents and frequencies) was used to detect DA release in the NAcc of each rat.

#### Pharmacological validation

Pharmacological validation took place 11–20 days after test day. DA and noradrenaline are indistinguishable by FSCV (Heien et al., 2003). Therefore, to further characterize the electrochemical signal, rats (*n* = 3, one rat was removed due to electrode instability) were subjected to acute tests with systemically-administered drugs. The following drugs were used: the D2/D3 agonist (-)-quinpirole hydrochloride (Sigma Aldrich, Oakville, ON), the DAT blocker GBR12909 2HCl (NIMH Chemical Synthesis and Drug Supply Program), the D2/D3 antagonist raclopride, the α2-adrenoreceptor antagonist yohimbine (Tocris Bioscience, Minneapolis, MN), the NET blocker desipramine hydrochloride (Sigma-RBI, St. Louis, MO) and D-amphetamine (Sigma-Aldrich, Poole, UK). Not all drugs were tested in each rat; see the Results Section and Supplementary Table 1 for details. All doses are expressed as the salt and all drugs were administered by the IP route (see Supplementary Table 2 for drug details). All drugs were dissolved in 0.9% sterile saline except for: GBR12909 in dimethyl sulfoxide (DMSO) and yohimbine in distilled water. The timing and pH of each control (vehicle) injection matched that of the respective drug. Drugs were administered in a volume of 1 mL/kg except for GBR12909 (2 mL/kg).

#### Histological verification of recording sites

DA is the predominant electroactive neurotransmitter within the NAcc and more specifically, in the lateral core (Garris et al., 1994) and rostral shell subregions (Park et al., 2010); thus, our microsensor targeted these regions. At the end of the experiment, rats were deeply anesthetized with IP ketamine (100 mg/kg) and xylazine (20 mg/kg) and an electrolytic lesion (+0.8 mA, 20 s, direct current) was made to facilitate histological identification. Rats were transcardially perfused with saline and then a 10% aqueous formalin solution (Sigma, St. Louis, MO). The brain was then removed and flash-frozen at −50°C with 2-methylbutane (Acros Organics, New Jersey, USA). Brains were cut on a cryostat (25-μm coronal sections, −20°C) and mounted on glass microscope slides. Sections were stained with cresyl violet to aid visualization of anatomical structures. When visible, the microsensor tip was localized by following the tract made by the fused-silica shaft.

## Experiment 2 optogenetic stimulation of DAergic neurons

### Subjects

Seven heterozygote tyrosine hydroxylase (TH)::Cre males rats (Witten et al., 2011) from Dr. Shizgal's colony were used. These rats originated from a generous donation from Dr. Karl Deisseroth (Stanford University) and Dr. Ilana Witten (Princeton University), and were subsequently outbred with Long-Evans rats (Charles River Laboratories, St. Constant, Quebec, Canada). Rats weighed 350–400 g at surgery. Subjects were singly housed in a temperature and humidity-controlled colony room (20–22°C, 50–60%) and were kept on a reverse 12:12 h light/dark cycle, with lights off at 0730 h. Behavioral testing took place between 0800 and 1600 h. Food and water were available *ad libitum*, except during testing. All procedures were carried out in accordance with the requirements of the Canadian Council on Animal Care and with the approval of the Concordia University Animal Research Ethics Committee.

### Rationale for viral construct use

Our optogenetic approach allowed us to selectively stimulate midbrain DA neurons to fire transiently (Tsai et al., 2009; Britt et al., 2012). We achieved neurochemical specificity by using genetically engineered rats that express Cre recombinase under the control of the tyrosine-hydroxylase promoter (Tsai et al., 2009; Witten et al., 2011). Cre-inducible viral constructs coding for channelrhoposin-2 (ChR2) were injected into the VTA (see Section Stereotaxic Virus Injections and Optical Fiber Implantation below), where cell bodies that give rise to ascending DAergic projections are located. In the genetically engineered rats employed, DA neurons express Cre and thus will express ChR2 following viral infection. Blue light was then used to activate ChR2, which leads to membrane depolarization and action-potential generation in DA neurons (see Section Optical Stimulation below). This light-driven neuronal activation produces phasic DA release in terminal regions (Bass et al., 2010; McCutcheon et al., 2014; Melchior et al., 2015).

### Stereotaxic virus injections and optical fiber implantation

Rats were first anesthetized with a solution of ketamine (87 mg/kg, Bionicle, Bellville, Ontario) and xylazine (13 mg/kg, Bayer Inc., Toronto, Ontario), given IP in a volume of 1 mL/kg, followed by atropine sulfate (0.02–0.05 mg/kg, 1 mL/kg, SC, Sandoz Canada Inc., Quebec) to reduce bronchial secretions. Polyvinyl alcohol (1% w/v, “HypoTears” Novartis) was applied to the eyes, and penicillin procaine G (300 000 IU/mL, 0.3 mL, SC, Bimeda-MTC Animal Health Inc., Cambridge, Ontario) was used as a prophylactic antibiotic. Under isoflurane anesthesia (5% + O_2_ for induction, 2–2.5% + O_2_ for maintenance), the skull was exposed by scalpel incision and six small burr holes were drilled above target coordinates. For injections of virus [AAV-DIO-ChR2-EYFP, University of North Carolina (UNC) Vector Core facility], a 28 gauge injector was lowered into the VTA [anterior-posterior (AP) −5.4 and −6.2 mm; medial-lateral (ML) ± 0.7 mm] and 0.5 μL of virus was infused at a rate of 0.1 μL/min at each of three dorsal-ventral (DV) coordinates (−8.2, −7.7, and −7.2 mm) for each of the two AP coordinates per hemisphere, for a total of 12 injections per brain. Virus was allowed to diffuse for 10 min between each injection. The total injection volume of virus was 6 μL (3 μL/hemisphere: three DV coordinates for each of the two AP coordinates). Chronically implantable optic fibers with a 300 μm core and a 0.37 numerical aperture were constructed following the methods described by Sparta et al. (2012). Optical fibers were implanted bilaterally into the VTA at a 10° angle sloping laterally from the vertical (AP −5.8 mm; ML ± 0.7 mm; DV −8.12 and –8.02 mm). Skull holes were covered with Gelfoam™ (Upjohn Company of Canada, Don Mills, Ontario) and the optical implants were anchored with a combination of skull screws and dental acrylic. Animals were given the opioid analgesic buprenorphine (0.05 mg/kg SC, 1 mL/kg, RB Pharmaceuticals Ltd., Berkshire, UK) and returned to their home cages for recovery. Behavioral manipulations began 5–7 weeks later to allow for viral construct expression.

### Optical stimulation

The light source for the optical stimulation was a 473 nm, 150 mW, diode-pumped, solid-state laser (Shanghai Laser and Optics Century Company, Shanghai, China). The output of this laser was routed to the chronically implanted optical fiber via a laser-to-fiber coupler (Oz Optics, Ottawa, ON, Canada), two optical patch cords, and an optical rotary joint (Doric Lenses, Quebec, QC, Canada). A patch cord (0.22 NA, 62.5 um core/125 um cladding, 3 mm reinforced jacket; AFL Global, Duncan, SC, USA) linked the output of the coupler to the rotary joint, which was mounted at the top of the test chamber; a robust, custom-built patch cord (0.39 NA; 200 μm core/225 μm cladding/500 μm buffer; Trujillo-Pisanty et al., 2015) fed the output of the rotary joint to the implanted fiber. This arrangement allowed the rat to circle freely. A pulse stimulator (A.M.P.I, Master-9, Jerusalem, Israel) was used to generate 1-s trains of 5 ms rectangular pulses of blue light. Each rat received optogenetic stimulation of VTA neurons through one of the implanted optical fibers; the optical probe used for stimulation (right or left) and optimal stimulation frequency were determined for each rat (see Section Optical Self-Stimulation Training below). The laser was allowed to reach its most stable operating temperature for 45–120 min before each training or test day. Continuous optical output from the custom-built patch cord was measured daily using a power meter (Thorlabs PM100D, Newton, NJ, USA) and kept at 40 ± 2 mW across sessions.

### Experimental protocol

Experiment 2 comprised the following four phases: (1) Optogenetic self-stimulation training, (2) USV screen, (3) Fixed interval training and (4) Testing on multiple schedules.

#### Optogenetic self-stimulation training

First, each rat (*n* = 7) was screened in order to optimize the optical stimulation, by determining: (1) which optical probe (i.e., left or right) was more effective behaviorally (as described next), and (2) the optimal pulse frequency, defined as the lowest optical pulse frequency that supported a maximal rate of self-stimulation. To determine the most effective probe, a successive approximation procedure was used (Peterson, 2004). Briefly, one optical implant was connected, and rats were trained to lever press on a fixed-ratio 1 schedule (FR1; one response required to receive stimulation) for unilateral delivery of 1-s trains of 5-ms light pulses (473 mW, 60 Hz) into the VTA. Once the animal acquired the lever pressing behavior, two 15-min FR1 trials began. Both trials began with the lever retracted, and their extension was announced by a 10-s flashing light situated above the test box. A single depression of the extended lever triggered a stimulation train and initiated a 2-s blackout period during which the lever was retracted. The total number of lever presses during the 15-min sessions was counted. Next, the contralateral optical probe was tested the following day in the same way. The probe that supported the most pressing (i.e., most presses in a 15 min session) was considered most effective and was used for the rest of the experiment. In some rats, both probes produced similar behavioral outputs. When this happened, both optical probes were retested on a third day and the most effective one from that session was retained. Next, to determine the optimal pulse frequency, rats were permitted to lever-press for unilateral optical stimulation of the VTA, on a 2-s cumulative handling time schedule (Breton et al., 2009). In this schedule, rats earned one train of VTA stimulation (1-s duration, 5-ms light pulses) for every 2 s of hold-down time (i.e., a total of 2 s which could be accumulated over one or more presses). Each trial was announced by a 10-s flashing light. At 2 s before the start of the trial, a non-contingent priming stimulation was delivered which matched the pulse frequency of the contingent stimulation offered in that trial. The pulse frequency was systematically decremented across ten 2-min trials to obtain a sigmoidal response rate vs. pulse-frequency curve. The pulse frequencies varied across subjects but typically ranged from 2 Hz (lower asymptote) to 60 Hz (higher asymptote). Between two and five sessions were required to construct a reliable rate-frequency curve. The lowest pulse frequency that yielded maximal responding was considered optimal. In all subsequent testing, the pulse frequency was held at the optimal value for a given rat (pulse range: 28–59, for details see Supplementary Table 3).

#### USV screen

Near the end of optical self-stimulation training, rats were screened for USV emission in a single session. Acoustic recordings began when rats were removed from their home cage and continued for 2–5 min during optical self-stimulation training in their test box. As expected (Schwarting et al., 2007; Wright et al., 2010; Scardochio and Clarke, 2013), a subset of rats (2/7) emitted few vocalizations (< 5 calls/min) and were therefore excluded from the experiment, leaving a total of 5 experimental subjects.

#### Fixed-interval (FI) training

The rats were next trained to respond on a fixed-interval (FI) schedule. On such a schedule, the lever is armed after a fixed delay. In our implementation, the first response after the delay elapsed triggered delivery of a stimulation train and restarted timing of the delay. Training began with the delay set to 1 s (FI-1) and progressed until the rat performed reliably with a 20 s delay (FI-20). Three to 12 daily sessions, each lasting 20 min, were run. The criterion for lengthening the delay during training was the emergence of the scalloped pattern of responding characteristic of performance on FI schedules (accelerated pressing as the end of the delay approached).

#### Testing on multiple schedules

Over the course of 8 consecutive days, each rat was tested on four different 20-s schedules of optogenetic stimulation, i.e., fixed and variable interval (FI-20, VI-20), and fixed and variable time (FT-20, VT-20). Each schedule was tested in two test sessions (1 session per day) lasting 2 h. Schedules were tested in the following order: FI-20, FT-20, VI-20, and VT-20. Under the FI and FT schedules, the stimulation was *expected*, insofar as it was delivered (FT) or became available (FI) every 20 s. Under VI and VT schedules, the stimulation was always *unexpected*, because the time between consecutive stimulations was sampled from an exponential distribution. To prevent scheduling of a subsequent reward before the optical stimulation had terminated, a fixed, 1.2 s lag was incorporated in the exponential distributions. Lever presses were recorded and reward delivery was programmed using software written in LabVIEW (National Instruments). Under fixed and variable *interval* schedules, the lever was always extended. After expiration of the minimum interval, a single lever press triggered the optical stimulation train; prior to this, lever presses were without effect. For fixed and variable *time* schedules, the stimulation was delivered non-contingently, and no lever was present. The interval length (20 s) and the test session duration (2 h) were selected based on previous work (Chehayeb, 2007; Ludvig et al., 2007). USVs were recorded for the whole session. No discrete cues (e.g., light or tone) were present and testing was performed in a dark room illuminated only with a red light. Since the rats virtually stopped calling within 30 min (see Section Results), only the first 30 min of calls and lever-pressing behavior were analyzed in detail.

### Histology

Immunohistochemistry was performed to assess the localization of optical fibers with respect to the spread of infection; colocalization of yellow fluorescent protein (YFP) and tyrosine hydroxylase (TH) was used to confirm the expression of the viral construct (YFP-ChR2) in DAergic neurons (TH-positive). Hence, upon completion of *in vivo* experiments, rats were transcardially perfused with 4% paraformaldehyde in phosphate-buffered saline (PBS). Brains were removed and post-fixed in a 4% paraformaldehyde/30% sucrose solution for 2 days, and kept frozen at −20°C thereafter. 40 μm sections mounted on Superfrost Plus™ microscope slides (Thermo Fisher Scientific, Ottawa, Ontario), washed with PBST (phosphate buffered saline with Triton: 0.1M PBS + 0.3% Triton X-100) for 1 min (× 3), and incubated with 10% normal donkey serum (Sigma Aldrich, Oakville, ON) in PBST for 30 min, followed by a 1 min wash in PBS. To assess the spread and confirm the selectivity of expression of the viral construct, anti-TH and anti-GFP immunohistochemistry was performed. YFP, a variant of GFP, is readily recognized by GFP antibodies (Vashist, 2013). Slices were incubated for 48 h with primary antibodies diluted in 10% donkey serum-PBST solution at the following concentrations: mouse anti-GFP (1:1000, Invitrogen, #A11120) and rabbit anti-TH (1:100, Fisher, #AB152MI). Slices were washed with PBS (3 × 5 min) and incubated at room temperature for 24 h with the following secondary antibodies: donkey anti-mouse AlexaFluor 488 and donkey anti-rabbit AlexaFluor 594 (both 1:200, Jackson Immuno, West Grove, PA, USA). Slides were washed for 5 min (× 3), tissues were allowed to dry and then slides were coverslipped with Vectashield® mounting medium with DAPI (Vector Laboratories, Inc., Burlingame, CA, USA). Sections were subsequently imaged (4, 20, and 60X) on a Nikon Livescan Sweptfield confocal microscope to determine colocalization of TH and the ChR2-YFP construct. High magnification images were reconstructed along the z axis by means of the 3D view, image snapshot and image adjustment tools in Imaris Scientific Software (Bitplane, Concord, MA). Non-linear gamma adjustments were conducted to show detail in high magnification images.

## Results

### Experiment 1: MFB stimulation and rat ultrasonic vocalizations

One rat was removed from all analyses because its electrical connection failed intermittently (hence, *n* = 3 rats).

### Initial amphetamine screen

The median 50-kHz call rate on the third day of the AMPH screen was 76 calls per min (range 53–102, *n* = 4 rats). Based on previous studies (Wright et al., 2010; Ahrens et al., 2013; Scardochio and Clarke, 2013), all the rats would be classified as “high callers” (i.e., above-average call rate).

### Ultrasonic vocalizations following electrical stimulation of the MFB

**Figure 1:**
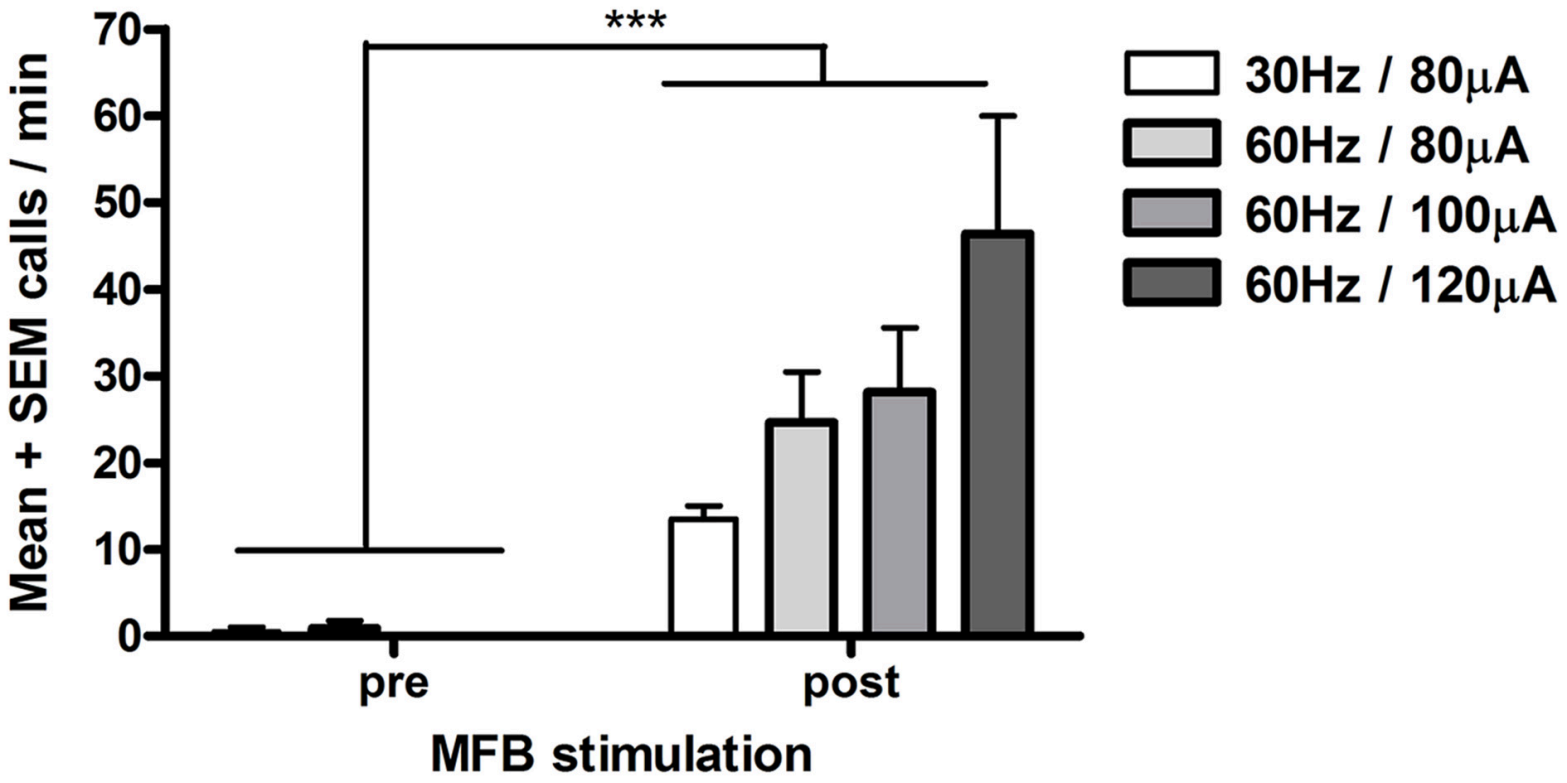
**Electrical stimulation of the medial forebrain bundle (MFB) and 50-kHz call rate (Experiment 1)**. Each rat (*n* = 3) was tested under each stimulation condition. Calls after MFB stimulation (post, 0–55 s following stimulation onset) were significantly greater than calls before MFB stimulation (pre, 0–55 s before stimulation). Each stimulation train was spaced 5–6 min apart. ^***^p < 0.002 pre vs. post (Sign test, *n* = 12 i.e., 4 stimulation parameters × 3 rats).

**Figure 2:**
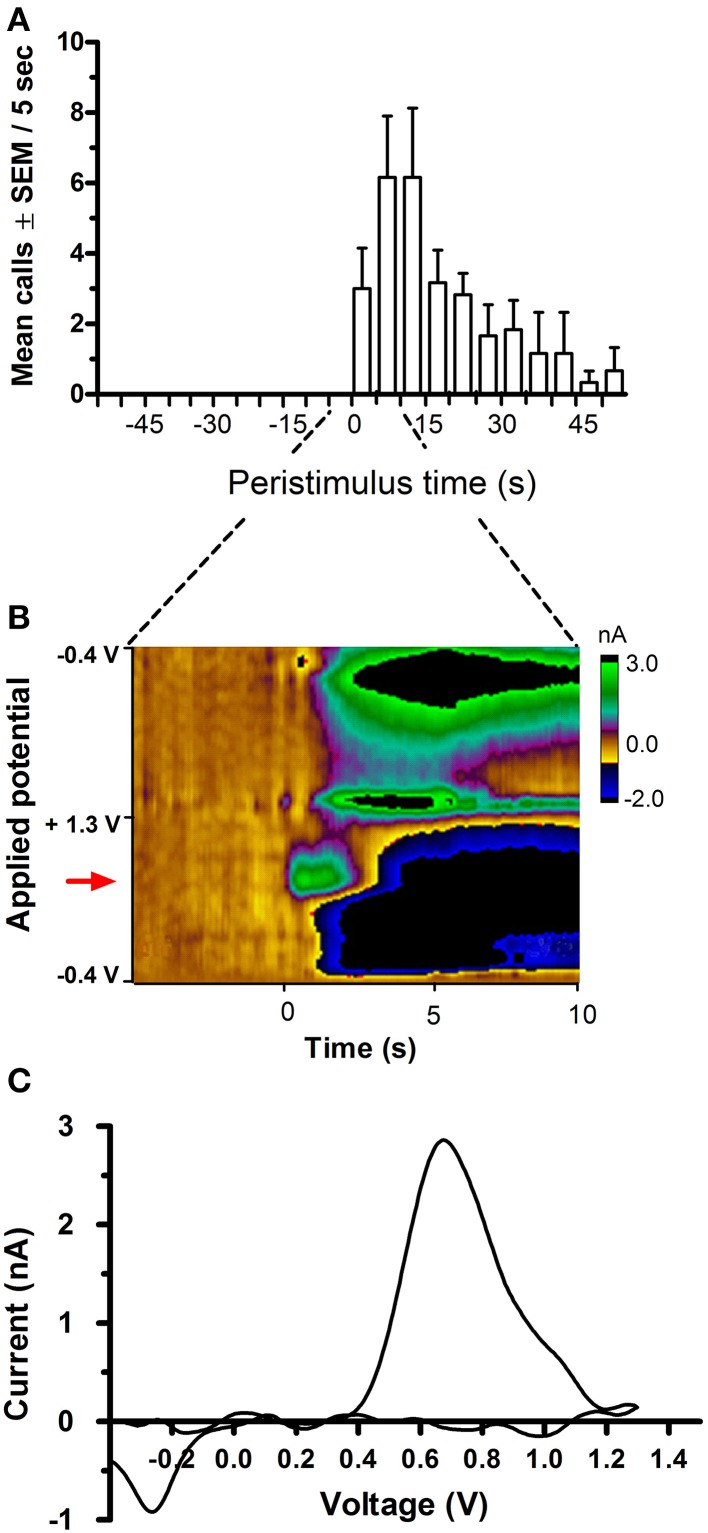
**Time course of 50-kHz call emission and phasic DA release following electrical stimulation of the medial forebrain bundle (MFB)**. Stimulated DA release in the nucleus accumbens (NAcc) was timed-locked to MFB stimulation (24 biphasic 120 μA 60 Hz pulses, each pulse comprising a pair of 2-ms phases) and the onset of USV emission. **(A)** shows an increase in call rate following MFB stimulation. The 1-s stimulation started at 0 s. **(B)** is a false-color plot from a representative rat, showing changes in DA current (green color) in relation to applied potential (y-axis) and time (x-axis), with onset of MFB stimulation occurring at *t* = 0 s, at the oxidation potential of dopamine (red arrow), i.e., ~0.65 V (vs. Ag/AgCl reference). **(C)** shows the corresponding background-subtracted cyclic voltammogram from the same rat at the point of peak DA current seen in **(B)**.

Electrical stimulation of the MFB significantly increased 50-kHz call rate (*n* = 3 rats, Sign test, *p* < 0.002, Figure 1). Calls were evoked promptly, starting ~1 s from the start of each stimulation train (Figure 2). The main call subtypes emitted following MFB stimulation were trill, flat/trill combo and flat (Supplementary Figure 1), which accounted for 42, 27, and 13% of all calls, respectively (Supplementary Figure 2). In one rat, more intense stimulation parameters were tested (60 Hz, 140 μA), producing 22-kHz as well as 50-kHz calls; therefore, these stimulation parameters were not tested with the other rats.

### Phasic DA release in the nucleus accumbens following electrical stimulation of the MFB

Data presentation is restricted to the five electrodes confirmed to be located in the NAcc and recording phasic DA release; only one electrode was excluded (see Section Characterization of *in vivo* Voltammetric Signals above).

**Figure 3:**
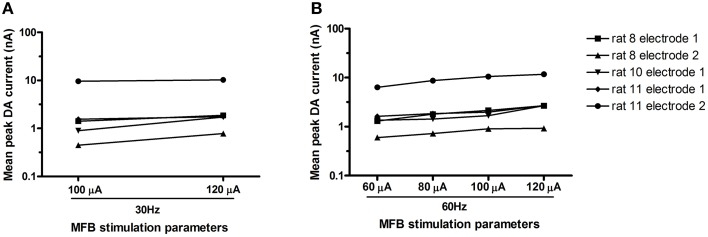
**Mean peak DA current (log) for each electrode (***n*** = 5 electrodes) across various stimulation parameters**. At both frequencies tested [30 Hz–**(A);** 60 Hz–**(B)**], peak DA current increased with current (respectively: Wilcoxon, *p* < 0.05; Friedman, *p* = 0.002).

Unilateral electrical stimulation of the MFB evoked phasic dopamine release in the NAcc (Figure 2). The evoked release was bilateral (*n* = 2 rats, data not shown). Mean peak DA currents increased with increasing applied current, for both stimulation frequencies, i.e., 30 Hz (Wilcoxon, *p* < 0.05) and 60 Hz (Friedman, *p* = 0.002; Figure 3). MFB stimulation rapidly increased the call rate (Figure 2A) and the DA signal (Figure 2B), with both effects appearing within ~1 s of stimulation onset. In contrast, the vocalization response far outlasted the transient DA signal increase. A subset of stimulations (32% of total stimulations) produced an increase in phasic DA in the NAcc without evoking an increase in call rate (for a summary of results for each rat under each stimulation parameter, see Supplementary Table 4).

### Characterization of *in vivo* voltammetric signals

#### Electrically-evoked release

Following stimulation of MFB fibers, DA was detected at almost all recording sites, as revealed by background-subtracted CVs (Figures 2B,C). The exception was microsensor (i.e., electrode) #2 from rat 10, which was excluded from all analyses (thus, *n* = 5 electrodes).

#### Pharmacological verification

The peak DA current evoked by electrical stimulation increased significantly following an acute administration of: the indirect DA/NA agonist amphetamine (paired *t*-test *p* < 0.05); the D2 antagonist raclopride (paired *t*-test, *p* < 0.05); and the DAT blocker GBR12909 (Supplementary Figure 3, paired *t*-test, *p* < 0.02). The three other pharmacological conditions could not be assessed statistically because each drug was tested in a single rat; this is because two of the three rats died prematurely, one day after an injection of desipramine/yohimbine, and the other under anesthesia while its headcap was being repaired. See Supplementary Table 1 for details of completed drug conditions for each rat.

#### Histology

*Five* electrode tips were confirmed to lie within the NAcc (Supplementary Figure 4). One electrode location could not be verified and the corresponding data are not presented; this was the same electrode (#2 of rat 10) where electrically-evoked catecholamine release was not obtained.

### Experiment 2: optogenetic stimulation of midbrain dopaminergic neurons

**Figure 4:**
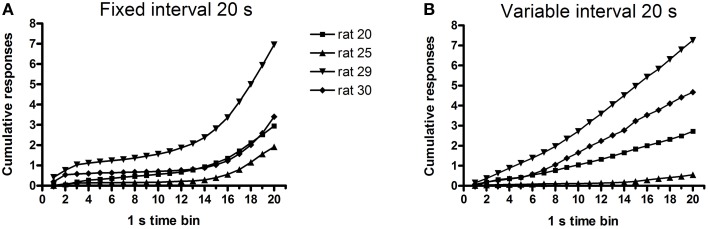
**Cumulative lever responses for each rat (***n*** = 4) as a function of time since the last optogenetic stimulation**. **(A)** shows lever responses increasing as the next stimulation opportunity approached (at 20 s). **(B)** shows lever pressing at a near-constant rate during the 10 s prior to the onset of the next stimulation opportunity (times randomly selected from lagged exponential distribution with mean 20 s).

**Figure 5:**
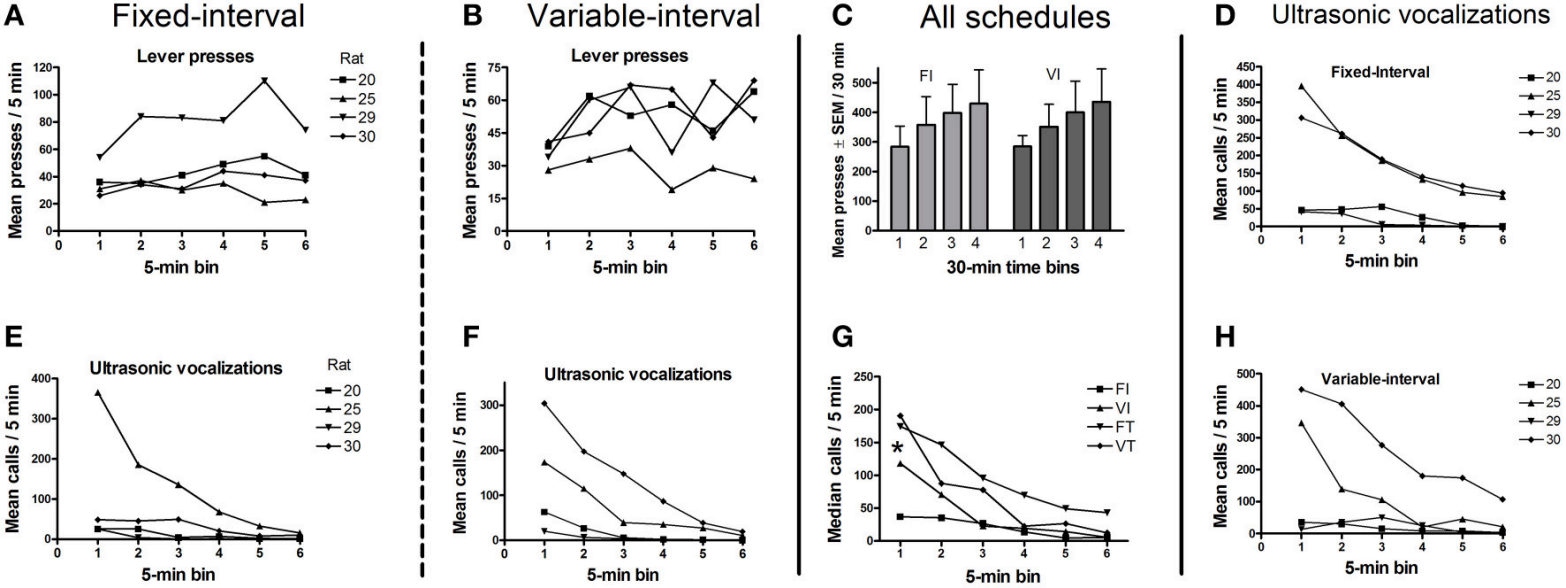
**Mean lever presses and mean calls for each schedule (***n*** = 4 rats)**. **(A,B)** show that lever press rates under both schedules remained stable across successive 5-min time bins within the first 30 min of the session. **(C)** shows that response rates tended to increase across the 2-h session. **(D–F,H)** show mean calls across all four reinforcement schedules, decreasing over the first 30 min of the for each rat. **(G)** shows that the median call rate decreased over the first 30 min for all schedules, with rats pooled. There was also a significant increase in call number when the optogenetic stimulation was non-contingent (VT and FT schedules) *vs*. contingent on a lever-press (FI and VI schedules). FI, fixed interval; VI, variable interval; FT, fixed interval; VT, variable interval. All schedules are 20 s. Sign-test ^*^p < 0.02 (*n* = 8 i.e., 4 rats × 2 schedules).

Histological verification confirmed that the virus was confined to the VTA and the substantia nigra pars compacta (see Section Histology below for details); hence, we refer below to the targets of the optical stimulation as midbrain DAergic neurons. One of the five rats in this experiment did not emit any USVs on test days, despite having done so during the screening session, and was thus excluded from the analysis. The remaining four rats worked to obtain trains of optogenetic stimulation on both FI-20 and VI-20 test schedules. As anticipated, responding was schedule-dependent. Thus, rats pressed faster toward the end of the 20-s fixed interval, whereas they maintained a steady response rate during the variable interval when the timing of the stimulation was unpredictable (Figure 4). Within the first 30 min of the test session, response rates were maintained (Figures 5A,B) whereas calls rate steadily declined (Figures 5D–H). Over the rest of the 2-h session, lever-press rates tended to increase (Figure 5C), but 50-kHz calling remained virtually undetectable (mean 1 ± 0.9 calls/min, not shown).

### USV emission: call numbers, categories, and timing with optogenetic stimulation

**Figure 6:**
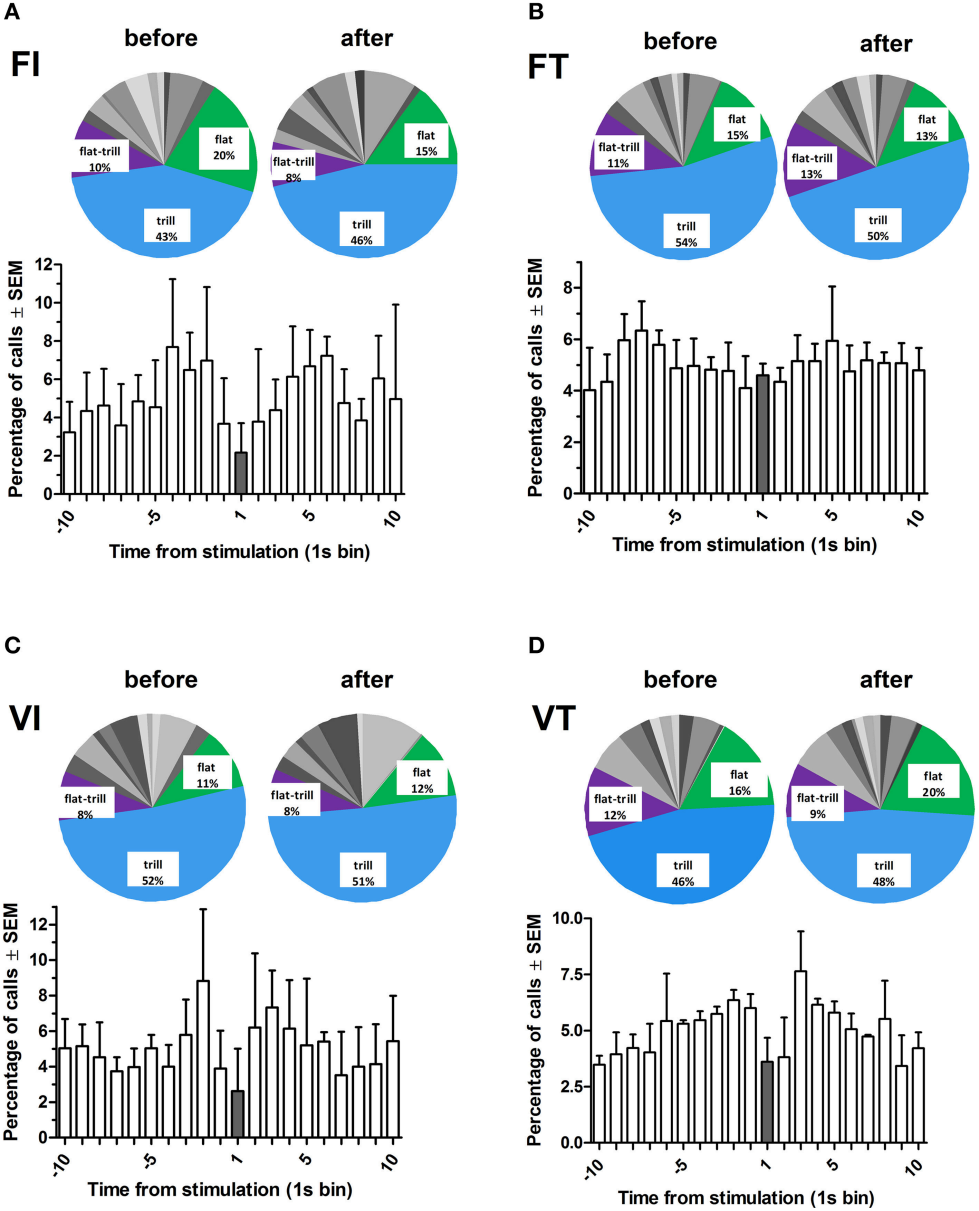
**Percentage of total calls and call subtypes under each reinforcement schedule for all rats (***n*** = 4), as a function of time from stimulation**. Time bin “1” represents the time at which the midbrain optogenetic stimulation occurred. Pie charts show percentages of calls (14 subtypes and 2 categories) before (–10 to –1) and after (2–10) the stimulation. The most common calls are labeled (for details of call subtypes see Supplementary Table 5). FI, fixed interval; FT, fixed time; VI, variable interval; VT, variable time. All schedules are 20 s.

Rats called at a broadly similar rate before and after each stimulation (Figure 6). Thus, call rates occurring in the 10 s before or after the stimulation did not differ significantly on any of the four schedules (Wilcoxon test, NS for each schedule). However, rats called less during the 1-s train of optogenetic stimulation under all schedules except fixed time (Figure 6). Overall, call rates were significantly higher on “time” schedules than on “interval” schedules (Figure 5G, Sign test, *p* < 0.02).

Call profiles (i.e., percentage prevalence of 14 call subtypes and two call categories) are shown in Figure 6 (insets) and Supplementary Figure 5. These profiles were comparable across the four schedules, and also between the 10-s periods before and after each stimulation (Friedman, NS). Overall, the most prevalent call subtypes were trills, flats, and flat-trills accounting for 49, 15, and 10%, respectively (For further details see Supplementary Table 5).

#### Assessment of habitual responding: supplementary experiment

To test whether responding had become habitual, four additional rats were tested in extinction (Supplementary Figure 7). When the laser light source was turned off after 1 h of self-stimulation, subjects abruptly stopped lever pressing, and promptly resumed responding when the optical stimulation was reinstated. This experiment used more intense optogenetic stimulation parameters than in Experiment 2 (Supplementary Figure 5 legend).

### Histology

Rats were sacrificed between 2 and 6 months after the last experimental test day. In all rats (*n* = 4), TH immunolabelling was considerably more intense in the VTA on the stimulated side of the brain, within approximately 170 μm of the end of the optical probe (Supplementary Figure 6). This asymmetry was apparent throughout the anterior-posterior extent of the VTA/SNC in each rat.

## Discussion

The present study provides two main novel findings. First, unexpected electrical stimulation of the MFB elicited both phasic DA release in the NAcc (lasting ~2 s) and a longer-lasting increase in 50-kHz vocalizations. Second, neurochemically-selective optogenetic stimulation of midbrain DAergic neurons, although reinforcing, did not consistently elicit 50-kHz calls.

### Electrical stimulation of the MFB produced concurrent USV emission and phasic DA release in the NAcc

In Experiment 1, unexpected electrical stimulation of the MFB elicited near *time-locked* 50-kHz vocalizations. In contrast, 50-kHz calling was previously reported shortly *after* electrical stimulation (Burgdorf et al., 2007), whereas in an earlier study, the same authors reported anticipatory calling (~5 s *before* stimulation) in response to non-contingent electrical stimulation (Burgdorf et al., 2000). At least two factors could contribute to the different temporal relationships between calls and stimulation trains observed here and in the two prior studies. First, Burgdorf et al. (2000) may have missed a considerable number of calls by using a heterodyne bat detector, since this device can only detect a subset of 50-kHz calls falling within its narrow frequency band. Second, our stimulation parameters differ considerably from those used in the two earlier reports.

In earlier studies (Burgdorf et al., 2000, 2007), evoked DA release was inferred from the observed behavior (i.e., reinforced responding), whereas we have directly measured it. Here, vocalizations co-occurred with electrically-evoked phasic DA release in the NAcc, but with exceptions. Notably, during a significant minority of trials (32%), electrical stimulation elicited phasic DA release but did not elicit any 50-kHz USVs. During these trials, peak DA release was not noticeably lower, suggesting that calls are not simply triggered when a specific threshold of peak concentration is achieved. In this context, it is worth noting that electrical stimulation of the MFB releases many other transmitters, including 5-HT, acetylcholine, glutamate, and noradrenaline, which all appear to modulate the emission of USVs (Brudzynski and Bihari, 1990; Wintink and Brudzynski, 2001; Wright et al., 2012; Wöhr et al., 2015).

### Disappearance of USVs despite continued optical stimulation

Despite continuing optogenetic stimulation of midbrain DAergic neurons, 50-kHz calls gradually disappeared over the first 30 min of the 2-h session. There are several possible explanations for this divergence. A trivial explanation would be that the larynx, which generates USVs (Johnson et al., 2010), became over-used and no longer capable of producing calls. However, this is most unlikely since adult rats can sustain high 50-kHz call rates (>50 calls/min) for at least an hour after acute amphetamine administration (Scardochio and Clarke, unpublished). A second possibility is that with repeated optogenetic stimulation, phasic DA transmission decreases and eventually becomes insufficient to support USV production. Three sets of observations support this notion. First, postsynaptic DA receptors may undergo desensitization, as has been observed in studies using electrical stimulation or pharmacological manipulations (Beaulieu and Gainetdinov, 2011). This appears unlikely given the persistence of optogenetic self-stimulation, a behavior which depends critically on phasic DA release (Bass et al., 2010, 2013; McCutcheon et al., 2014) and on functional D1 and D2 receptors in the NAcc (Steinberg et al., 2013, 2014). Second, intracranial self-stimulation studies have shown that intense neuronal activation can deplete the pool of releasable DA (Garris et al., 1999; Kilpatrick et al., 2000; Park et al., 2013). While we cannot exclude the possibility that the chronic optical stimulation applied here would have depleted vesicular DA, the observed lever-pressing behavior is nonetheless consistent with DA release. A third mechanism by which DA transmission might be reduced is through desensitization of channelrhodopsin-2 following intense optogenetic stimulation (Bamann et al., 2008; Lórenz-Fonfría and Heberle, 2014). A final and related possibility that we considered is that by 30 min into the session, the self-stimulation behavior had become habitual and thus insensitive to the outcome, i.e., evoked DA release. However, the results do not support this hypothesis. Consistent with observations by Witten et al. (2011), responding was clearly dependent on whether or not it triggered the optical stimulation. The steady responding seen prior and following the disconnection of the lever (Supplementary Figure 7) further suggests that prolonged stimulation in Experiment 2 did not lose its effectiveness in activating DAergic neurons (i.e., ChR2 was not desensitized).

### Call profile following optogenetic stimulation of midbrain DAergic neurons

Typically, 50-kHz vocalizations are analyzed as a single group of calls or are dichotomized as frequency-modulated and flat subtypes (Portfors, 2007). However, 50-kHz calls are far more heterogeneous, with at least 14 identifiable subtypes (Wright et al., 2010). During optogenetic stimulation sessions, the most commonly emitted call was the trill subtype. Among 50-kHz call subtypes, it is the trill call that is preferentially suppressed by DA-depleting lesions and DA antagonist administration (Ciucci et al., 2009; Wright et al., 2013), two manipulations which would be expected to reduce both tonic and phasic DA transmission. The preponderance of trill calls in optical stimulation sessions suggests that this call subtype is promoted by phasic DA release.

Frequency-modulated calls, and trills in particular, occur in situations where positive affect putatively occurs. For example, these calls are especially abundant in the following experimental situations: (1) acute systemic administration of psychostimulants (Ahrens et al., 2009; Wright et al., 2012; Scardochio and Clarke, 2013; Simola et al., 2014), (2) anticipated delivery of food or drug delivery (Buck et al., 2014a,b; Opiol et al., 2015), (3) rough-and-tumble play (Knutson et al., 1998), and (4) sexual behavior (Snoeren and Ågmo, 2014). Since we have shown that reinforced lever pressing can be dissociated from USV emission, it appears that trill calls, insofar as they track positive affect, do so independently of positive reinforcement.

### Limitations

#### Pharmacology

In Experiment 2, several pharmacological verification tests were planned in order to confirm the neurochemical identity of the FSCV signal evoked by electrical stimulation. However, as detailed in Results, three drug tests were curtailed because of unforeseen adverse events. For example, the combination of yohimbine and desipramine appeared highly toxic, even though the same dose combination has been used previously without apparent ill effect (Park et al., 2010). Despite the restricted pharmacological testing, two additional factors indicate that our FSCV signal would have been minimally contaminated by extracellular NA: first, our recording electrodes were located in parts of the NAcc that receive little or no NA innervation (Park et al., 2010) and second, the recording electrodes used here are reportedly more sensitive to DA than NA (Clark et al., 2010).

#### DA release

The hypothesis investigated in this study concerns only *phasic* DA release, which was measured directly in Experiment 1. Although phasic DA release during optogenetic stimulation of the VTA was not measured (in Experiment 2), it is very likely to have occurred for four main reasons. First, previous *in vitro* and *in vivo* studies have shown that optogenetic stimulation of VTA DAergic neurons reliably evokes phasic DA release in downstream targets such as the NAcc and dorsal striatum (Bass et al., 2010; Chiu et al., 2014). Second, there is minimal expression of ChR2 in neurons lacking tyrosine hydroxylase (Witten et al., 2011). Third, our histological verification confirms that the viral transfection was restricted to neurons that express tyrosine hydroxylase. Fourth, our preliminary recordings (Cossette et al., 2014) have confirmed that phasic DA release occurs in the NAcc following optogenetic stimulation of the VTA, using identical stimulation parameters.

#### Optogenetic test day order

In Experiment 2, the reinforcement schedules were tested in a fixed order, as follows: fixed-interval, fixed-time, variable-interval, and variable-time. This particular sequence was chosen so as to limit the number of training sessions after the acquisition of FR1 responding. Testing order, however, would not have acted as a significant confounder given that all four schedules dissociated persistent lever-pressing behavior from declining USV emission.

## Conclusion

We have confirmed that immediately following electrical stimulation of the MFB, rats start to emit 50-kHz calls. The onset of these vocalizations coincided with phasic DA release in the NAcc. Optical stimulation of midbrain DAergic neurons was initially associated with high 50-kHz call rates and a trill-rich call profile, as occurs after systemic administration of psychostimulants. However, because optogenetic stimulation of midbrain DAergic neurons did not produce sustained calling, experimentally-induced phasic DA release in the NAcc does not appear a *sufficient* stimulus to induce 50-kHz call emission.

## Author contributions

TS: Contributed to the conception and design of the work. Acquired and analyzed all data for Experiment 1. Acquired and analyzed the data for Experiment 2: USV screen, FI training, and testing on multiple schedules. Drafted and critically reviewed intellectual content. Submitted a final approval of the version to be published and is accountable for all aspects of the work. IT: Contributed to the conception and design of the work. Acquired and analyzed the data for Experiment 2: optogenetic surgeries, optical self-stimulation training, USV screen, training, and supervision on test days and histology. Critically reviewed and contributed to intellectual content. Submitted a final approval of the version to be published and is accountable for all aspects of the work. KC: Contributed to the content and analyzed data for Figure 4. Critically reviewed intellectual content. Submitted a final approval of the version to be published and is accountable for all aspects of the work. PS: Contributed to the conception and design of Experiment 2. Critically reviewed and contributed to intellectual content. Submitted a final approval of the version to be published and is accountable for all aspects of the work. PC: Contributed to the conception and design of both experiments. Drafted and critically reviewed intellectual content. Submitted a final approval of the version to be published and is accountable for all aspects of the work.

### Conflict of interest statement

The authors declare that the research was conducted in the absence of any commercial or financial relationships that could be construed as a potential conflict of interest.
