# Supplementary material for: The Effects of Electrical and Optical Stimulation of Midbrain Dopaminergic Neurons on Rat 50-kHz Ultrasonic Vocalizations
Source: Front Behav Neurosci. 2015 Dec 8;9:331. doi: 10.3389/fnbeh.2015.00331 (PMC4672056; doi:10.3389/fnbeh.2015.00331)
Supplement: Supplementary file 1 [file Table1.DOCX]

Supplementary Material

**The effects of electrical and optical stimulation of midbrain dopaminergic neurons on rat 50-kHz ultrasonic vocalizations**

Tina Scardochio^1^, Ivan Trujillo-Pisanty^2^, Kent Conover^2^, Peter Shizgal^2^, Paul B.S. Clarke^1,2^*

*** Correspondence:** Dr. Paul Clarke, paul.clarke@mcgill.ca

**Supplementary Table S1** Completed drug tests for each rat, used for the pharmacological validation of *in vivo* dopamine signals

| Rat ID | SAL | DMSO | AMPH | GBR | QUIN | RAC | DMI/YO | YO |
| --- | --- | --- | --- | --- | --- | --- | --- | --- |
| 8 | ✓ | ✓ | ✓ | ✓ | ✓ | ✓ |  | ✓ |
| 10 | ✓ | ✓ | ✓ | ✓ |  | ✓ | ✓ |  |
| 11 | ✓ | ✓ | ✓ | ✓ |  |  |  |  |

SAL (saline), DMSO (dimethyl sulfoxide), AMPH (amphetamine), GBR (GBR12909), QUIN (quinpirole), DMI (desipramine), YO (yohimbine), RAC (raclopride)
