# Supplementary material for: The Effects of Electrical and Optical Stimulation of Midbrain Dopaminergic Neurons on Rat 50-kHz Ultrasonic Vocalizations
Source: Front Behav Neurosci. 2015 Dec 8;9:331. doi: 10.3389/fnbeh.2015.00331 (PMC4672056; doi:10.3389/fnbeh.2015.00331)
Supplement: Supplementary file 2 [file Table2.DOCX]

Supplementary Material

**The effects of electrical and optical stimulation of midbrain dopaminergic neurons on rat 50-kHz ultrasonic vocalizations**

Tina Scardochio^1^, Ivan Trujillo-Pisanty^2^, Kent Conover^2^, Peter Shizgal^2^, Paul B.S. Clarke^1,2^*

*** Correspondence:** Dr. Paul Clarke, paul.clarke@mcgill.ca

**Supplementary Table 2** Summary of drugs used for the pharmacological verification of dopaminergic release

| **Treatment** | | | | | |  |
| --- | --- | --- | --- | --- | --- | --- |
| **Drug** | | **Dose**  **(mg/kg)** | **Route** | **Time before testing (min)** | **Anticipated effect on DA current** | **n (rats)** |
| D1/D2 agonist | quinpirole | 0, 0.3 | IP | 20 | Decrease | 1 |
| NET blocker + α2-adrenoreceptor antagonist | DMI + yohimbine | 0, 15 +  0, 6 |  | 20 | No change | 1 |
| α2-adrenoreceptor antagonist | yohimbine | 0, 6 |  | 20 | No change (or small increase) | 1 |
| DAT blocker | GBR12909 | 0, 15 |  | 30 | Increase | 3 |
| D2/D3 antagonist | raclopride | 0, 2 |  | 30 | Increase | 2 |
| Indirect DA/NA agonist | amphetamine | 0, 1.6 |  | -- | Increase | 3 |
