# Supplementary material for: The Effects of Electrical and Optical Stimulation of Midbrain Dopaminergic Neurons on Rat 50-kHz Ultrasonic Vocalizations
Source: Front Behav Neurosci. 2015 Dec 8;9:331. doi: 10.3389/fnbeh.2015.00331 (PMC4672056; doi:10.3389/fnbeh.2015.00331)
Supplement: Supplementary file 3 [file Table3.DOCX]

Supplementary Material

**The effects of electrical and optical stimulation of midbrain dopaminergic neurons on rat 50-kHz ultrasonic vocalizations**

Tina Scardochio^1^, Ivan Trujillo-Pisanty^2^, Kent Conover^2^, Peter Shizgal^2^, Paul B.S. Clarke^1,2^*

*** Correspondence:** Dr. Paul Clarke, paul.clarke@mcgill.ca

**Supplementary Table 3** Optimized stimulation parameters identified for each rat following optical self-stimulation training

|  | **Frequency** | |
| --- | --- | --- |
| **Rat ID** | # Pulses | Period (ms) |
| BeChR20 | 57 | 17.86 |
| BeChR25 | 51 | 19.90 |
| BeChR28 | 28 | 35.70 |
| BeChR29 | 34 | 29.41 |
| BeChR30 | 59 | 16.95 |
