# Supplementary material for: The Effects of Electrical and Optical Stimulation of Midbrain Dopaminergic Neurons on Rat 50-kHz Ultrasonic Vocalizations
Source: Front Behav Neurosci. 2015 Dec 8;9:331. doi: 10.3389/fnbeh.2015.00331 (PMC4672056; doi:10.3389/fnbeh.2015.00331)
Supplement: Supplementary file 4 [file Table4.DOCX]

Supplementary Material

**The effects of electrical and optical stimulation of midbrain dopaminergic neurons on rat 50-kHz ultrasonic vocalizations**

Tina Scardochio^1^, Ivan Trujillo-Pisanty^2^, Kent Conover^2^, Peter Shizgal^2^, Paul B.S. Clarke^1,2^*

*** Correspondence:** Dr. Paul Clarke, paul.clarke@mcgill.ca

**Supplementary Table 4** Summary of MFB stimulation parameters tested for each rat with DA transient and USV emission results

|  | **Stimulation parameters** | | | | | | | | | | |
| --- | --- | --- | --- | --- | --- | --- | --- | --- | --- | --- | --- |
|  | **30 Hz** | | | | | **60 Hz** | | | | | |
| **Rat** | **40 µA** | **60 µA** | **80 µA** | **100 µA** | **120 µA** | **40 µA** | **60 µA** | **80 µA** | **100 µA** | **120 µA** | **140 µA** |
| **8** |  |  |  |  |  |  |  |  |  |  |  |
| **10** |  |  |  |  |  |  |  |  |  |  |  |
| **11** |  |  |  |  |  |  |  |  |  |  |  |

**Not tested**

**DA and USVs**

**Only USVs**

**Only DA**

**No DA or USVs**

* 50- and 22-kHz calls. Note: each cell represents one stimulation trial
