# Supplementary material for: The Effects of Electrical and Optical Stimulation of Midbrain Dopaminergic Neurons on Rat 50-kHz Ultrasonic Vocalizations
Source: Front Behav Neurosci. 2015 Dec 8;9:331. doi: 10.3389/fnbeh.2015.00331 (PMC4672056; doi:10.3389/fnbeh.2015.00331)
Supplement: Supplementary file 5 [file Table5.DOCX]

Supplementary Material

**The effects of electrical and optical stimulation of midbrain dopaminergic neurons on rat 50-kHz ultrasonic vocalizations**

Tina Scardochio^1^, Ivan Trujillo-Pisanty^2^, Kent Conover^2^, Peter Shizgal^2^, Paul B.S. Clarke^1,2^*

*** Correspondence:** Dr. Paul Clarke, paul.clarke@mcgill.ca

**Supplementary Table 5** Call percentages (14 subtypes and 2 categories) before and after optogenetic stimulation of midbrain DAergic neurons, under four reinforcement schedules

| **SCHEDULE** | **TIMING** | **RAT** | **T** | **F** | **FT** | **CX** | **MS** | **C** | **UR** | **DR** | **SU** | **SD** | **TJ** | **SP** | **S** | **IU** | **M** | **UN** |
| --- | --- | --- | --- | --- | --- | --- | --- | --- | --- | --- | --- | --- | --- | --- | --- | --- | --- | --- |
| **FI** | Before | **20** | 43 | 17 | 7 | 15 | 4 | 0 | 0 | 4 | 2 | 0 | 4 | 0 | 2 | 2 | 0 | 0 |
|  | After |  | 41 | 23 | 2 | 14 | 0 | 0 | 0 | 7 | 0 | 0 | 7 | 0 | 0 | 2 | 0 | 4 |
| **FT** | Before |  | 20 | 34 | 10 | 8 | 2 | 0 | 0 | 0 | 10 | 3 | 5 | 0 | 4 | 1 | 0 | 3 |
|  | After |  | 20 | 35 | 9 | 6 | 5 | 0 | 1 | 4 | 6 | 2 | 4 | 1 | 2 | 2 | 1 | 2 |
| **VI** | Before |  | 24 | 28 | 5 | 12 | 6 | 0 | 0 | 7 | 3 | 1 | 5 | 1 | 5 | 2 | 0 | 1 |
|  | After |  | 35 | 28 | 7 | 11 | 9 | 0 | 0 | 2 | 0 | 0 | 1 | 0 | 5 | 0 | 0 | 2 |
| **VT** | Before |  | 19 | 40 | 12 | 6 | 3 | 1 | 0 | 3 | 4 | 2 | 4 | 0 | 2 | 3 | 0 | 1 |
|  | After |  | 26 | 47 | 3 | 3 | 2 | 0 | 0 | 3 | 3 | 2 | 5 | 2 | 2 | 0 | 0 | 2 |
| **FI** | Before | **25** | 43 | 12 | 20 | 3 | 5 | 4 | 0 | 0 | 5 | 2 | 2 | 0 | 2 | 1 | 0 | 1 |
|  | After |  | 47 | 12 | 20 | 2 | 4 | 3 | 1 | 0 | 5 | 2 | 1 | 0 | 2 | 0 | 0 | 1 |
| **FT** | Before |  | 55 | 7 | 19 | 3 | 2 | 2 | 1 | 0 | 4 | 1 | 3 | 0 | 1 | 1 | 0 | 1 |
|  | After |  | 52 | 7 | 21 | 3 | 2 | 3 | 0 | 0 | 5 | 1 | 3 | 0 | 1 | 1 | 0 | 1 |
| **VI** | Before |  | 56 | 4 | 15 | 7 | 3 | 2 | 0 | 0 | 3 | 3 | 3 | 0 | 2 | 1 | 0 | 1 |
|  | After |  | 47 | 11 | 18 | 5 | 5 | 0 | 0 | 0 | 4 | 4 | 3 | 0 | 2 | 1 | 0 | 0 |
| **VT** | Before |  | 49 | 7 | 14 | 2 | 2 | 6 | 1 | 0 | 2 | 2 | 13 | 0 | 1 | 1 | 0 | 0 |
|  | After |  | 53 | 7 | 13 | 2 | 1 | 6 | 1 | 1 | 2 | 0 | 14 | 0 | 0 | 0 | 0 | 0 |
| **FI** | Before | **29** | 20 | 44 | 8 | 4 | 8 | 0 | 0 | 4 | 4 | 0 | 0 | 0 | 4 | 4 | 0 | 0 |
|  | After |  | 23 | 18 | 5 | 18 | 0 | 0 | 0 | 0 | 9 | 9 | 0 | 5 | 8 | 5 | 0 | 0 |
| **FT** | Before |  | 58 | 9 | 8 | 10 | 1 | 1 | 0 | 1 | 5 | 1 | 0 | 0 | 3 | 2 | 0 | 1 |
|  | After |  | 49 | 8 | 11 | 7 | 1 | 0 | 0 | 1 | 7 | 1 | 0 | 0 | 7 | 6 | 0 | 2 |
| **VI** | Before |  | 47 | 10 | 7 | 7 | 0 | 3 | 0 | 3 | 10 | 0 | 0 | 0 | 10 | 3 | 0 | 0 |
|  | After |  | 44 | 6 | 0 | 25 | 0 | 0 | 0 | 0 | 6 | 0 | 0 | 0 | 19 | 0 | 0 | 0 |
| **VT** | Before |  | 44 | 16 | 10 | 8 | 1 | 1 | 1 | 0 | 8 | 2 | 4 | 0 | 3 | 2 | 0 | 0 |
|  | After |  | 42 | 20 | 10 | 9 | 2 | 1 | 0 | 0 | 5 | 3 | 3 | 0 | 4 | 0 | 0 | 1 |
| **FI** | Before | **30** | 66 | 8 | 6 | 2 | 0 | 1 | 1 | 1 | 1 | 0 | 3 | 0 | 7 | 0 | 0 | 4 |
|  | After |  | 71 | 5 | 4 | 2 | 1 | 1 | 0 | 1 | 1 | 2 | 1 | 0 | 9 | 0 | 0 | 2 |
| **FT** | Before |  | 80 | 1 | 10 | 1 | 1 | 2 | 0 | 0 | 1 | 0 | 3 | 0 | 1 | 0 | 0 | 0 |
|  | After |  | 77 | 2 | 12 | 1 | 0 | 2 | 0 | 0 | 2 | 0 | 3 | 0 | 1 | 0 | 0 | 0 |
| **VI** | Before |  | 78 | 2 | 7 | 1 | 0 | 0 | 0 | 0 | 2 | 1 | 5 | 0 | 3 | 0 | 0 | 1 |
|  | After |  | 77 | 2 | 8 | 2 | 0 | 1 | 0 | 0 | 2 | 1 | 5 | 0 | 1 | 0 | 0 | 1 |
| **VT** | Before |  | 73 | 3 | 11 | 2 | 1 | 1 | 0 | 0 | 2 | 0 | 5 | 0 | 1 | 0 | 0 | 1 |
|  | After |  | 70 | 2 | 12 | 2 | 2 | 1 | 0 | 0 | 3 | 0 | 7 | 0 | 1 | 0 | 0 | 0 |

T: trill; F: flat; FT: flat-trill; CX: complex; MS: multistep; C: complex; UR: up-ramp; DR: down-ramp; SU: step-up; SD: step-down; TJ: trill-jump; SP: split; S: short; IU: inverted-U; M: miscellaneous; UN: unclassifiable (as described in Wright et al., 2010)
