# Supplementary material for: The Effects of Electrical and Optical Stimulation of Midbrain Dopaminergic Neurons on Rat 50-kHz Ultrasonic Vocalizations
Source: Front Behav Neurosci. 2015 Dec 8;9:331. doi: 10.3389/fnbeh.2015.00331 (PMC4672056; doi:10.3389/fnbeh.2015.00331)
Supplement: Supplementary file 6 [file DataSheet1.DOCX]

Supplementary Material

**The effects of electrical and optical stimulation of midbrain dopaminergic neurons on rat 50-kHz ultrasonic vocalizations**

Tina Scardochio^1^, Ivan Trujillo-Pisanty^2^, Kent Conover^2^, Peter Shizgal^2^, Paul B.S. Clarke^1,2^*

*** Correspondence:** Dr. Paul Clarke, paul.clarke@mcgill.ca


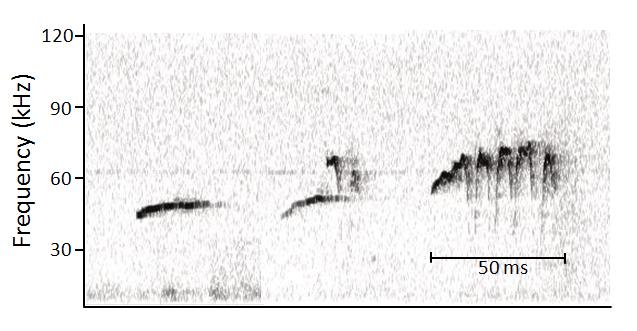


**Supplementary Figure 1** Example spectrogram from one rat, of the most commonly emitted 50-kHz calls following electrical stimulation of the MFB and optical stimulation of midbrain DAergic neurons. From left to right: flat, flat-trill and trill. Flat calls have a near constant frequency and a mean slope between -0.2 and 0.2 kHz/ms. Flat-trill combination calls are a trill flanked on one or both sides by a monotonic portion that is no less than 10 ms. Trill calls are rapid frequency oscillations with a period of approximately 15 ms (Wright et al. 2010).
