# Supplementary material for: The Effects of Electrical and Optical Stimulation of Midbrain Dopaminergic Neurons on Rat 50-kHz Ultrasonic Vocalizations
Source: Front Behav Neurosci. 2015 Dec 8;9:331. doi: 10.3389/fnbeh.2015.00331 (PMC4672056; doi:10.3389/fnbeh.2015.00331)
Supplement: Supplementary file 8 [file DataSheet3.DOCX]

Supplementary Material

**The effects of electrical and optical stimulation of midbrain dopaminergic neurons on rat 50-kHz ultrasonic vocalizations**

Tina Scardochio^1^, Ivan Trujillo-Pisanty^2^, Kent Conover^2^, Peter Shizgal^2^, Paul B.S. Clarke^1,2^*

*** Correspondence:** Dr. Paul Clarke, paul.clarke@mcgill.ca


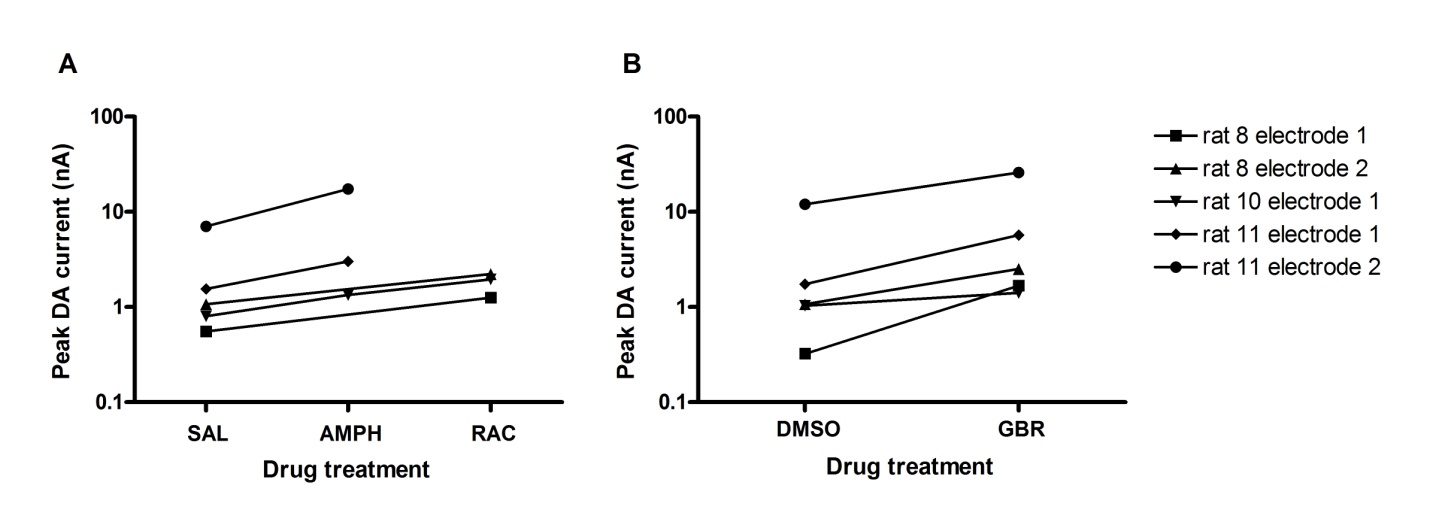


**Supplementary Figure 3** Pharmacological validation of dopaminergic release from voltammetric recordings in the nucleus accumbens. Panel **(A)** shows a significant increase in electrically-induced (24 biphasic 120 µA 60 Hz pulses, each pulse comprising a pair of 2-ms phases) peak DA current following an acute administration of the indirect DA/NA agonist amphetamine (AMPH) (paired t-test, p<0.05) and the D2 antagonist raclopride (RAC) (paired t-test, p<0.05). Panel **(B)** shows a significant increase in electrically-induced (same parameters as panel B) peak DA current following an acute injection of the DAT blocker GBR12909 (GBR) (paired t-test, p<0.02). Vehicle conditions: SAL (saline), DMSO (dimethyl sulfoxide).
