# Supplementary material for: The Effects of Electrical and Optical Stimulation of Midbrain Dopaminergic Neurons on Rat 50-kHz Ultrasonic Vocalizations
Source: Front Behav Neurosci. 2015 Dec 8;9:331. doi: 10.3389/fnbeh.2015.00331 (PMC4672056; doi:10.3389/fnbeh.2015.00331)
Supplement: Supplementary file 9 [file DataSheet4.DOCX]

Supplementary Material

**The effects of electrical and optical stimulation of midbrain dopaminergic neurons on rat 50-kHz ultrasonic vocalizations**

Tina Scardochio^1^, Ivan Trujillo-Pisanty^2^, Kent Conover^2^, Peter Shizgal^2^, Paul B.S. Clarke^1,2^*

*** Correspondence:** Dr. Paul Clarke, paul.clarke@mcgill.ca


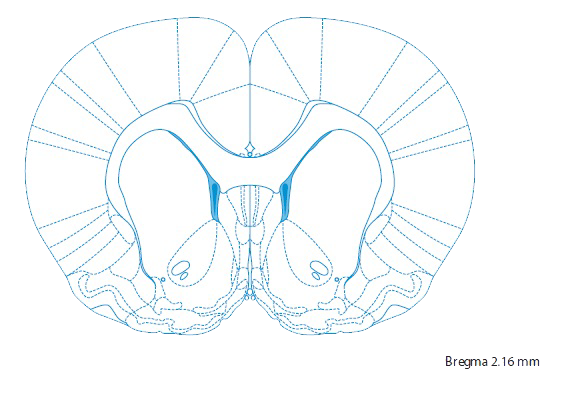

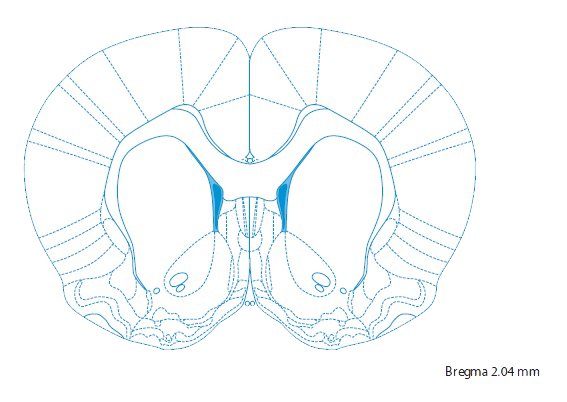

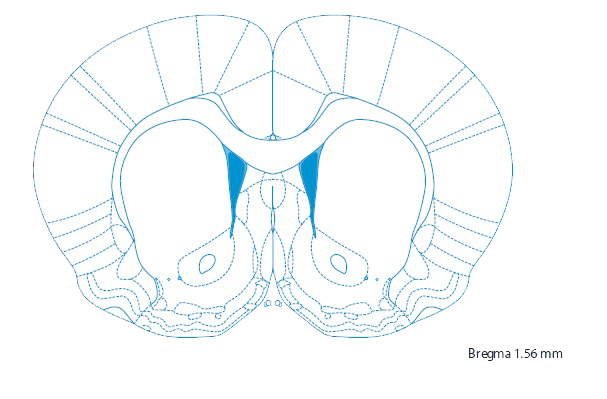


rat 10 electrode 1

rat 8 electrodes 1 & 2

rat 11 electrodes 1 &2

1.56 mm

2.04 mm

2.16 mm

**Supplementary Figure 4** Location of voltammetric microsensors (n=5). Each point represents the approximate center of a microsensor. Coordinates are in millimeters anterior to bregma and the coronal drawings are from the Paxinos and Watson (2007) atlas.

Paxinos, G., and Watson, C. (2007). The Rat Brain in Stereotaxic Coordinates, 6th Edn. New York: Academic Press.
