# Supplementary material for: The Effects of Electrical and Optical Stimulation of Midbrain Dopaminergic Neurons on Rat 50-kHz Ultrasonic Vocalizations
Source: Front Behav Neurosci. 2015 Dec 8;9:331. doi: 10.3389/fnbeh.2015.00331 (PMC4672056; doi:10.3389/fnbeh.2015.00331)
Supplement: Supplementary file 10 [file DataSheet5.DOCX]

Supplementary Material

**The effects of electrical and optical stimulation of midbrain dopaminergic neurons on rat 50-kHz ultrasonic vocalizations**

Tina Scardochio^1^, Ivan Trujillo-Pisanty^2^, Kent Conover^2^, Peter Shizgal^2^, Paul B.S. Clarke^1,2^*

*** Correspondence:** Dr. Paul Clarke, paul.clarke@mcgill.ca


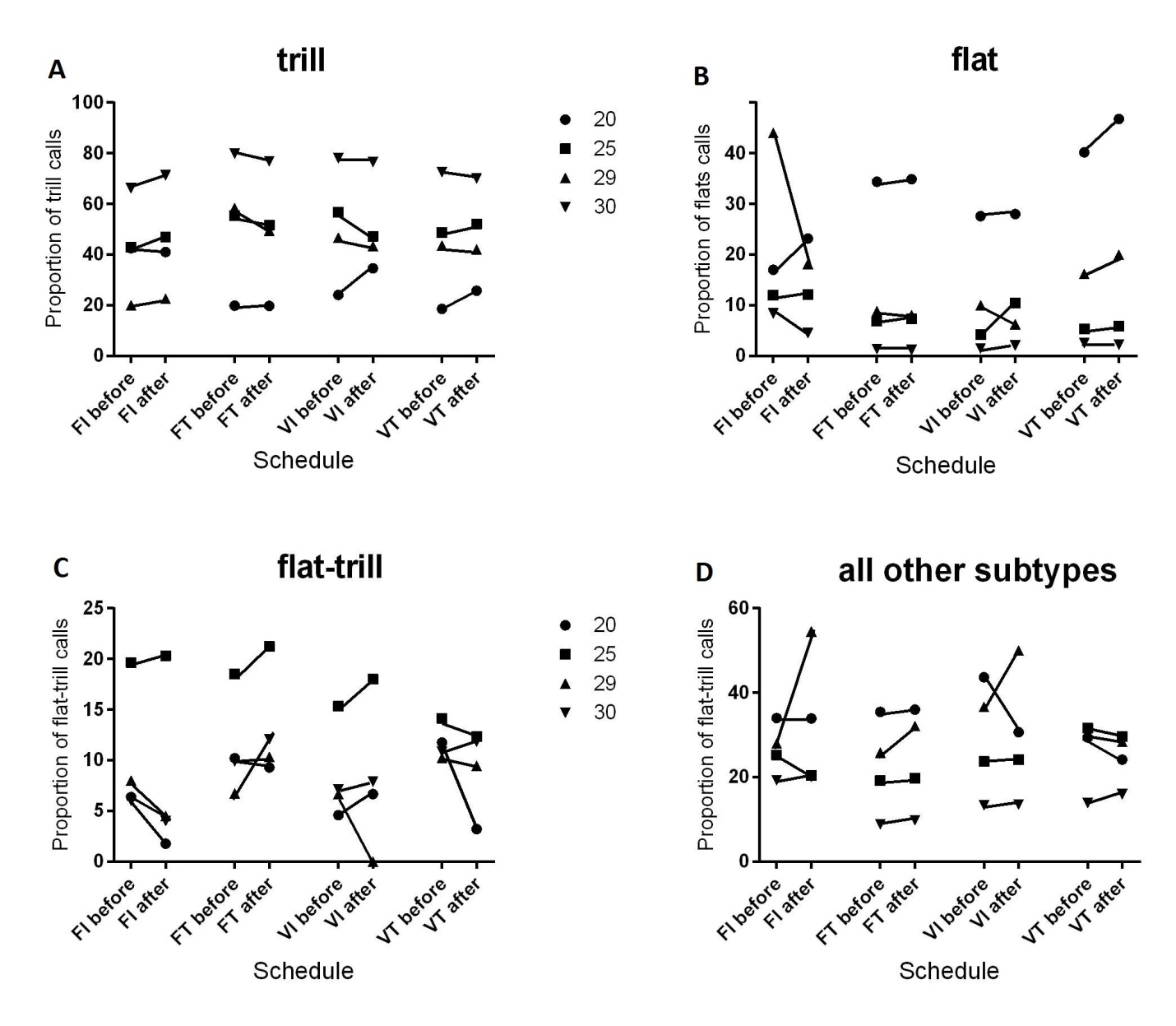


**Supplementary Figure 5** Proportions of 50-kHz call subtypes for each rat and for each reinforcement schedule, before and after optogenetic stimulation of midbrain DAergic neurons. Panels **(A)**, **(B)** and **(C)** show the proportions of trill, flat and flat-trill call subtypes, i.e. the three most common calls observed. Panel **(D)** is the total proportion of other calls pooled together (13 subtypes). FI: fixed interval, FT: fixed time, VI: variable interval, VT: variable time
