# Supplementary material for: The Effects of Electrical and Optical Stimulation of Midbrain Dopaminergic Neurons on Rat 50-kHz Ultrasonic Vocalizations
Source: Front Behav Neurosci. 2015 Dec 8;9:331. doi: 10.3389/fnbeh.2015.00331 (PMC4672056; doi:10.3389/fnbeh.2015.00331)
Supplement: Supplementary file 11 [file DataSheet6.DOCX]

Supplementary Material

**The effects of electrical and optical stimulation of midbrain dopaminergic neurons on rat 50-kHz ultrasonic vocalizations**

Tina Scardochio^1^, Ivan Trujillo-Pisanty^2^, Kent Conover^2^, Peter Shizgal^2^, Paul B.S. Clarke^1,2^*

*** Correspondence:** Dr. Paul Clarke, paul.clarke@mcgill.ca


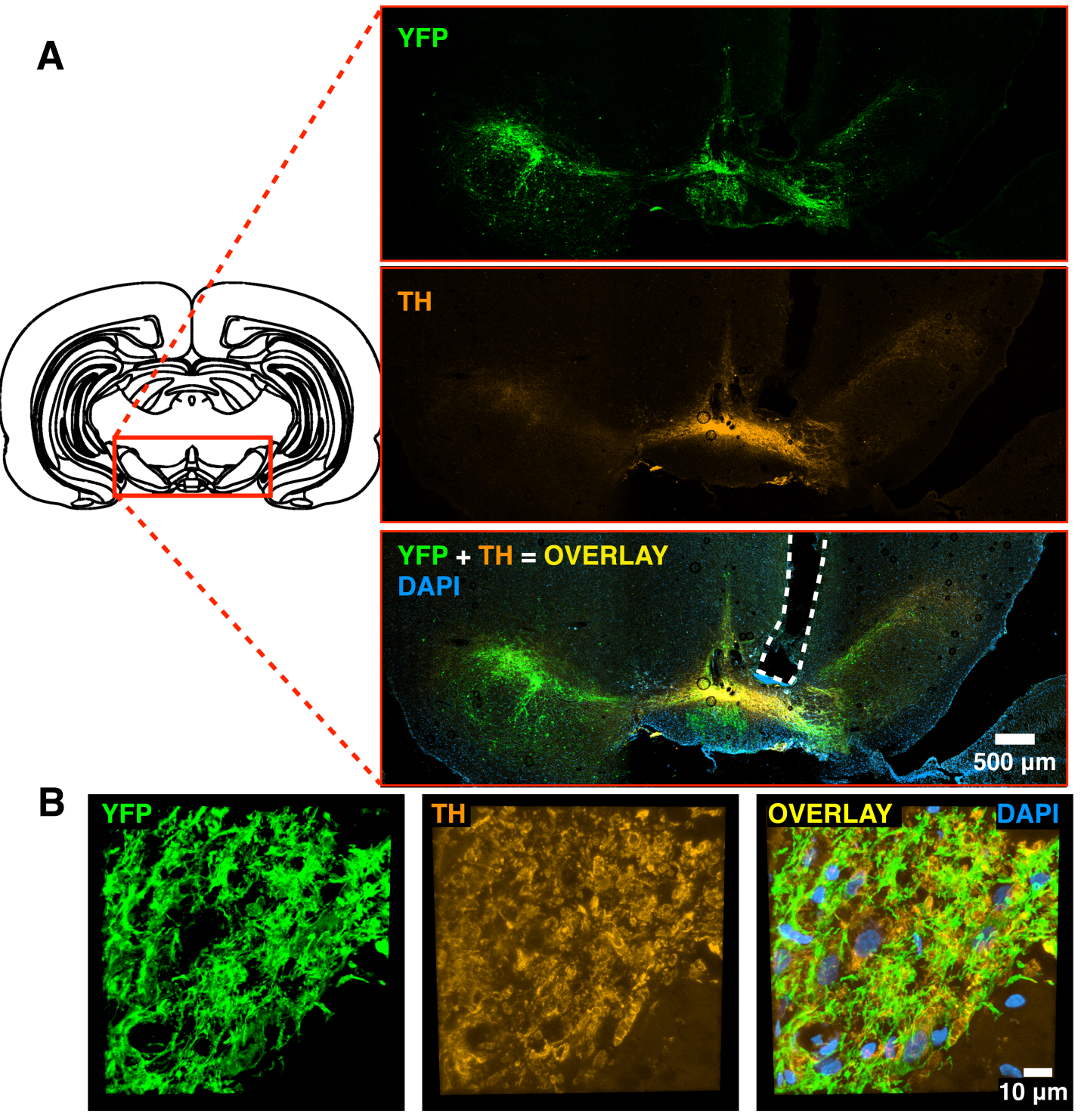


**Supplementary Figure 6** Spread of viral construct expression from one representative rat. **(A)** Left: Schematic representation of a coronal section at the approximate location of the optical implant tip (modified from Paxinos and Watson, 2007). Right: Representative immunohistochemistry images. YFP and TH are shown in the top and middle panels respectively. The bottom panel shows the co-expression of YFP and TH (overlay), along with DAPI for anatomical reference. The dashed line indicates the location of the optical implant **(B)** High magnification 3D reconstruction of the area below the optical fiber track. Left: YFP positive neurons; Middle: TH positive neurons; Right: YFP and TH overlay and DAPI. Note that YFP expression overlaps with TH positive neurons.

Paxinos, G., and Watson, C. (2007). The Rat Brain in Stereotaxic Coordinates, 6th Edn. New York: Academic Press.
