# Supplementary material for: The Effects of Electrical and Optical Stimulation of Midbrain Dopaminergic Neurons on Rat 50-kHz Ultrasonic Vocalizations
Source: Front Behav Neurosci. 2015 Dec 8;9:331. doi: 10.3389/fnbeh.2015.00331 (PMC4672056; doi:10.3389/fnbeh.2015.00331)
Supplement: Supplementary file 12 [file DataSheet7.DOCX]

Supplementary Material

**The effects of electrical and optical stimulation of midbrain dopaminergic neurons on rat 50-kHz ultrasonic vocalizations**

Tina Scardochio^1^, Ivan Trujillo-Pisanty^2^, Kent Conover^2^, Peter Shizgal^2^, Paul B.S. Clarke^1,2^*

*** Correspondence:** Dr. Paul Clarke, paul.clarke@mcgill.ca


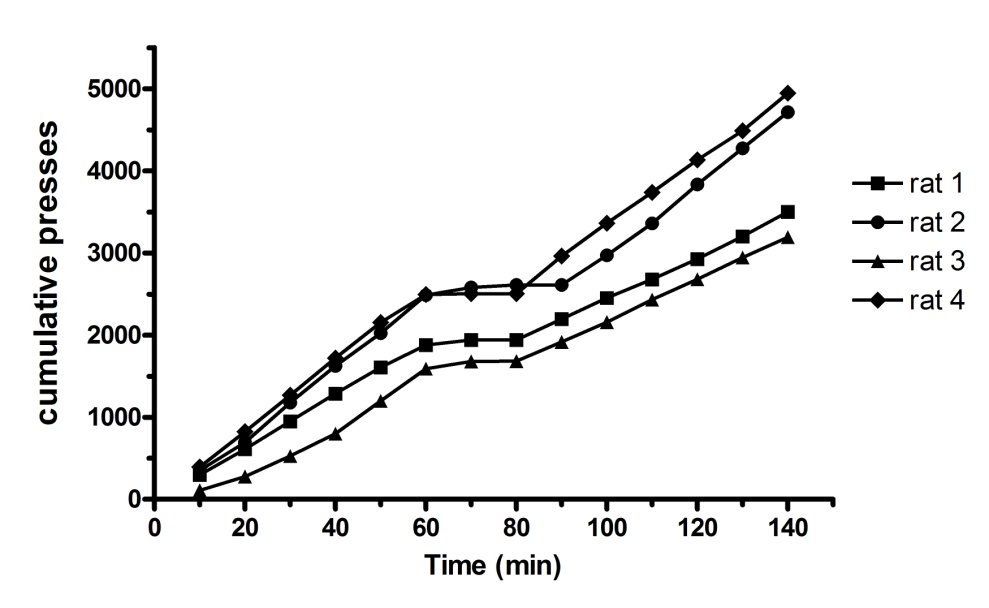


**Supplementary Figure 7** Cumulative lever presses during optogenetic self-stimulation of midbrain DAergic neurons. Rats (n=4) were trained to hold down a lever for 2 s (which could be accumulated across different hold-downs) to obtain a 1 s train of 5 ms light pulses into the VTA (28-56 Hz). A 2 s blackout period (lever retraction) followed each reward. The laser for optogenetic stimulation was turned on during the first 6 sessions (time 10-60), was turned off for the two next sessions (minutes 60-80) and subsequently turned back on. Rats only pressed when stimulation was available.
